# Design and Synthesis of Marine Sarocladione Derivatives with Potential Anticancer Activity

**DOI:** 10.3390/md24010048

**Published:** 2026-01-20

**Authors:** Xiao-Mei Liu, Wen-Xuan Li, Ling-Xiu Kong, Guan-Ying Han, Jinghan Gui, Xu-Wen Li

**Affiliations:** 1 State Key Laboratory of Chemical Biology, Shanghai Institute of Materia Medica, Chinese Academy of Sciences, Shanghai 201203, China; liuxiaomei@simm.ac.cn; 2 Shandong Laboratory of Yantai Drug Discovery, Bohai Rim Advanced Research Institute for Drug Discovery, Yantai 264117, China; wenxuanli2022@163.com (W.-X.L.); 19553596368@163.com (L.-X.K.); 3 University of Chinese Academy of Sciences, Beijing 100049, China; 4 Shandong Fengjin Biopharmaceuticals Co., Ltd., Yantai 264117, China; 5 State Key Laboratory of Chemical Biology, Shanghai Institute of Organic Chemistry, Chinese Academy of Sciences, Shanghai 200032, China; 6 Shanghai Academy of Natural Sciences (SANS), Shanghai 200031, China

**Keywords:** marine natural product, sarocladione, anticancer activity, apoptosis, SAR

## Abstract

The discovery of structurally novel anti-tumor agents remains a crucial objective in cancer drug research. In this study, we systematically explored the bioactivity potential of sarocladione (**5**), a structurally unique marine-derived 14-membered ring diketone steroid. Guided by a function-oriented strategy, seven new derivatives (**6**–**13**) were synthesized based on an efficient biomimetic synthesis of sarocladione. Evaluation of their antiproliferative activities against human cancer cell lines demonstrated that the intact macrocyclic scaffold is indispensable for activity. Extension of the conjugated π-system led to the identification of compound **8**, which exhibited approximately four-fold enhanced potency against HCT116 cells (IC_50_ = 1.86 µM) compared with the parent natural product. Stereochemical analysis further revealed the critical role of the (5R)-configuration at C-5. Phenotypic investigations indicated that compound **8** induces concentration-dependent G2/M phase cell cycle arrest, followed by apoptosis, suggesting a cell cycle-dependent antiproliferative effect. Overall, this study highlights sarocladione as a promising marine-derived scaffold for further antiproliferative optimization.

## 1. Introduction

Natural products have served as an inexhaustible source of inspiration and leads in drug discovery due to their unique chemical diversity and profound biological relevance [1,2,3]. This is exemplified by the fact that a significant proportion of approved drugs, especially in areas like anticancer and anti-infective therapies, are either natural products or derived from them [4,5,6,7]. In particular, marine natural products (MNPs) have emerged as a treasure trove for discovering new scaffolds with potent bioactivities, attributed to the extreme and competitive marine environments that drive the evolution of unique biosynthetic pathways [8]. The continuous discovery of structurally complex and bioactive MNPs from diverse marine organisms (e.g., sponges, corals, and microorganisms) underscores their immense potential [9,10,11]. Concurrently, our research team is dedicated to tapping into this rich marine resource, with a particular focus on marine-derived fungi as a source of structurally distinctive natural products and on the strategic modification of bioactive small molecules with druggable potential [12,13,14,15].

**Figure 1:**
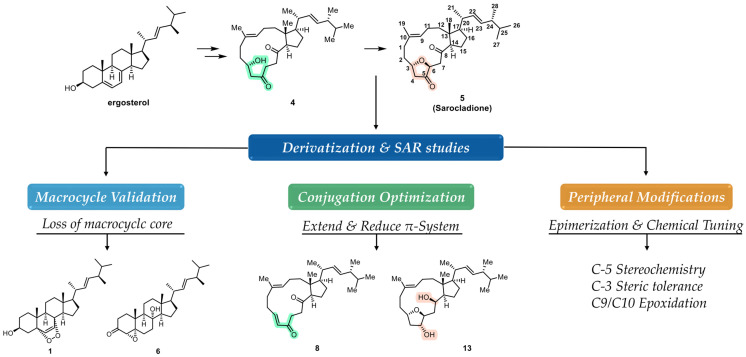
Function-oriented design strategy highlighting key structural elements of Sarocladione subjected to systematic modification for SAR exploration.

Sarocladione (**5**, Figure 1), a structurally unique 5,10:8,9-diseco-steroid featuring a 14-membered ring diketone moiety, represents such a new scaffold [16]. Its seco-steroid architecture is rare among natural products and often correlates with distinct biological profiles, as seen in other classes [17]. It was first isolated in 2012 from an endolichenic fungus *Sporormiella irregularis* [18], and later independently obtained from the deep-sea fungus *Sarocladium kiliense* in 2019, where it was formally named [16]. A significant breakthrough was achieved in 2021 by Gui and co-workers, who accomplished the first biomimetic synthesis of **5** starting from ergosterol in an exceptionally concise manner (2 or 7 steps) [19]. This elegant synthesis not only revised its structure but, more importantly, opened up a practical avenue for its systematic derivatization. Importantly, such biomimetic access provides a rare opportunity to translate a marine-derived, structurally unique natural product into a tractable platform for systematic structure–activity relationship (SAR) exploration [20,21].

Despite this synthetic achievement, the biological profile of sarocladione and its analogs remains largely unexplored. In its initial isolation report, it did not show significant cytotoxicity [16]. Intriguingly, in our hands, sarocladione (**5**) exhibited reproducible, albeit moderate, antiproliferative activity against human colorectal carcinoma (HCT116, RKO) and lung carcinoma (A549) cell lines (IC_50_ 7.29–12.64 µM, Table 1). More broadly, sarocladione belongs to the intriguing class of marine-derived seco-steroids, which have attracted considerable synthetic interest due to their complex, rearranged architectures [22,23,24,25]. Preliminary screenings have indicated anticancer potential for several members of this structural family. For instance, aplysiasecosterol A exhibited moderate cytotoxicity against HL-60 cells (IC_50_ = 16 µM) [26], and strophasterol A has been reported to inhibit the proliferation of various cancer cell lines by inducing apoptosis [27]. However, these studies have largely remained at the level of activity discovery and reporting. Crucially, a systematic medicinal chemistry campaign aimed at establishing detailed structure–activity relationships (SAR) and optimizing the anticancer potency for any specific seco-steroid scaffold has been notably absent. The combination of its synthetically tractable nature, unique 5,10:8,9-diseco-architecture, and our observed preliminary activity positions sarocladione as an ideal candidate to address this gap. Therefore, this study was undertaken to perform the first systematic SAR exploration of the sarocladione scaffold through rational design, synthesis, and biological evaluation of a focused library of derivatives, with the goal of identifying novel leads with enhanced anticancer potential.

Building upon the synthetic breakthrough, we sought to systematically evaluate sarocladione as an optimizable antiproliferative scaffold. Our design strategy was rationalized as follows (Figure 1): (i) The macrocyclic diketone is the most distinctive feature; thus, loss of this ring (compounds **1** and **6**) would probe its essentiality for bioactivity. (ii) The diketone moiety may present a potential pharmacophore; we aimed to extend its conjugation (compound **8**) or reduce it (compound **13**) to modulate electron density and planarity. (iii) The peripheral C-3 hydroxyl and C-9/C-10 alkene offer handles for steric and electronic perturbation (compounds **7** and **9**). (iv) The configuration at the newly generated C-5 center could be critical for spatial orientation; thus, both epimers (**10** and **11**) were prepared. Collectively, these designs were intended to systematically dissect the contribution of macrocyclic integrity, conjugation, peripheral substitution, and stereochemical orientation to the antiproliferative activity of sarocladione. Accordingly, we report the efficient synthesis of Sarocladione (**5**) and seven new derivatives (**6**–**13**), evaluation of their antiproliferative activities, and preliminary phenotypic investigations of the most active compound.

## 2. Results

### 2.1. Chemistry

**Scheme 1:**
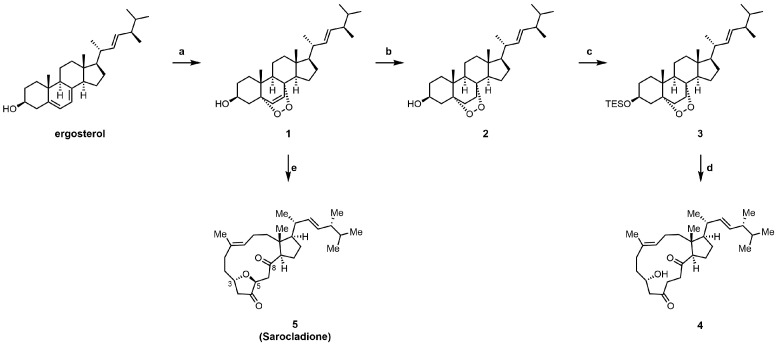
Synthesis of compounds **4** and **5**. Reagents and conditions: (a) O_2_, tetraphenylporphyrin (1 mol%), hv, CH_2_Cl_2_, 0 °C, 4 h, 66%; (b) potassium (*E*)-diazene-1,2-dicarboxylate (PADA) (4.1 equiv), HOAc (8.2 equiv), CH_2_Cl_2_/MeOH (1:2), 0 °C to rt, overnight, 99%; (c) TESCl (2.0 equiv), Im (4.0 equiv), DMAP (0.3 equiv), CH_2_Cl_2_, rt, 1 h, 95%; (d) [RuCl_2_(CO)_3_]_2_ (5 mol%), toluene, 150 °C (MW), 20 min; then HF•Py, Py, THF, rt, 15 min, 71%; (e) HOAc/H_2_O (100:1), reflux, 4 h, 15% or [RuCl_2_(CO)_3_]_2_ (5 mol%), toluene, 150 °C (MW), 20 min, 20%.

Following the established biomimetic route [19], we efficiently synthesized intermediate **4** and the natural product sarocladione (**5**) from ergosterol (Scheme 1). Using inexpensive and commercially available ergosterol as the starting material, a [4+2] reaction with singlet oxygen under photoirradiation yielded ergosterol endoperoxide **1**. Subsequent selective reduction of the alkene and protection with a silyl group afforded the saturated endoperoxide **3**. Compound **4** was then obtained via a ruthenium-catalyzed peroxygen fragmentation reaction. Alternatively, using ergosterol endoperoxide **1** as the substrate, a tandem peroxygen fragmentation/oxa-Michael addition sequence directly provided the natural product sarocladione (**5**).

**Scheme 2:**
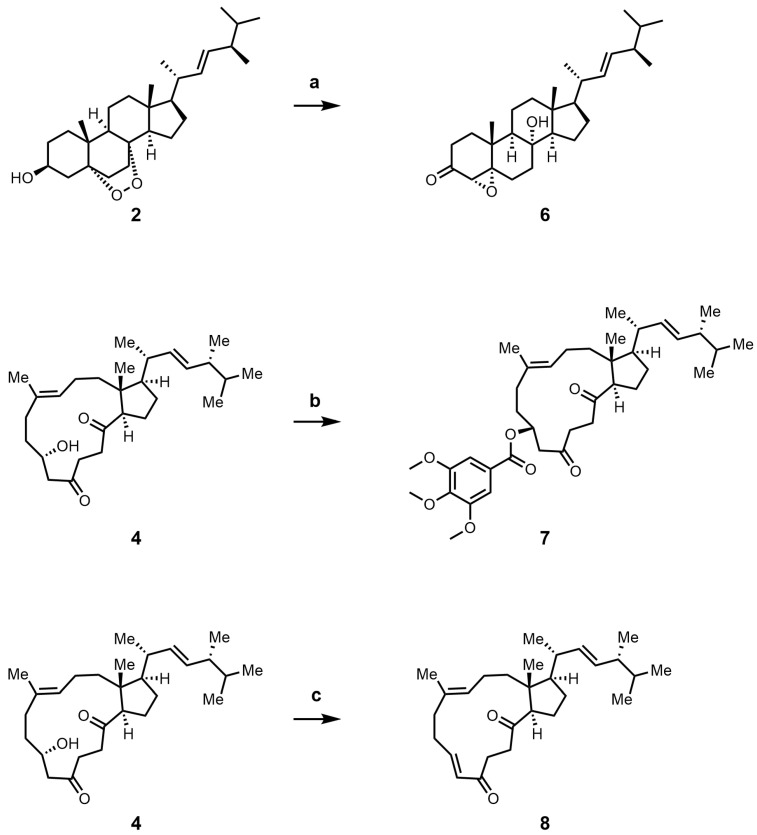
Synthesis of compounds **6**–**8**. Reagents and conditions: (a) Jones reagent (CrO_3_/H_2_SO_4_), acetone, rt, 1 h, 23%; (b) 3,4,5-trimethoxybenzoyl chloride (4 equiv), Et_3_N (8 equiv), DMAP (0.4 equiv), Py, rt, 2 h, 68%; (c) sulfamoyl chloride (2.0 equiv), DMA, rt, 3 h, 43%.

**Scheme 3:**
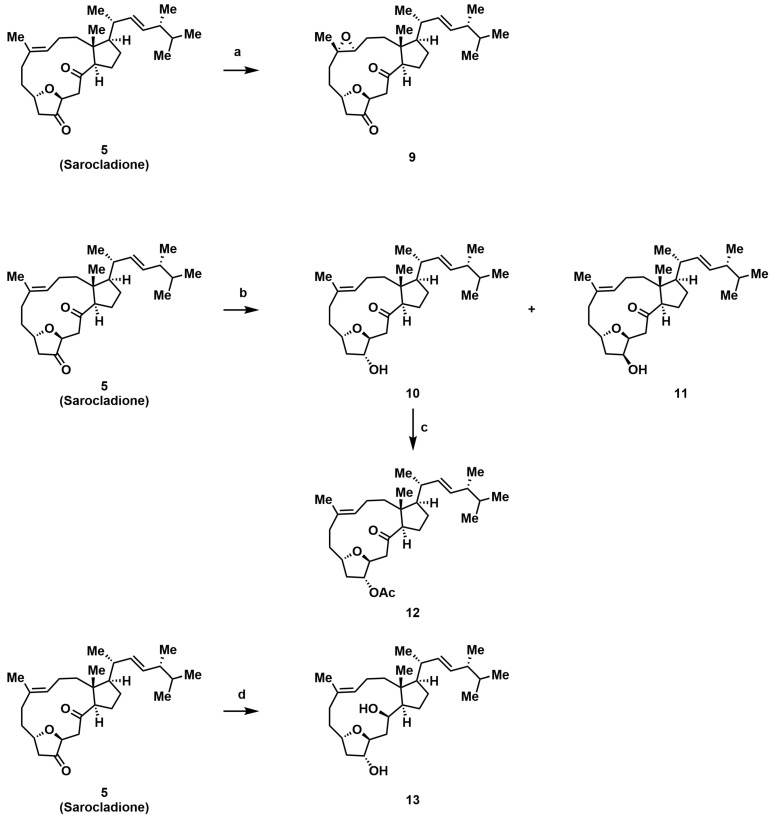
Synthesis of compounds **9**–**13**. Reagents and conditions: (a) *m*-CPBA (1.5 equiv), NaHCO_3_ (5 equiv), CH_2_Cl_2_, 0 °C, 0.5 h, 51%; (b) NaBH_4_ (0.5 equiv), THF, 0 °C, 1.5 h, 55% (for **10**), 20% (for **11**); (c) Ac_2_O (10 equiv), Py, CH_2_Cl_2_, rt, 1 h, 91%; (d) NaBH_4_ (2.0 equiv), THF, rt, overnight, 18%.

Guided by the design strategy outlined in the Introduction, we synthesized seven new derivatives (**6**–**13**) through targeted modifications of intermediates **4** and **5** (Scheme 2 and Scheme 3). To validate the necessity of the macrocyclic core, a comparison of the activities between the non-macrocyclic compound **1** and **6** against the intact natural product was conducted. To probe the effect of extending the conjugated π-system, we transformed compound **5** to introduce a C-3/C-4 double bond, yielding **8**. Conversely, full reduction of the C-5 and C-8 carbonyls of **5** gave compound **13**, which served as a control to assess the importance of the diketone functionality.

Attention was then turned to peripheral modifications. Acylation of the C-3 hydroxyl in compound **4** with 3,4,5-trimethoxybenzoyl chloride provided ester **7**, testing the steric tolerance at this site. Notably, attempts with bulkier or more electrophilic reagents (e.g., phthalic anhydride, sulfamoyl chloride) led exclusively to the dehydrogenated product **8**, highlighting a competing elimination pathway under forcing conditions. Epoxidation of the C-9/C-10 alkene in **5** yielded epoxide **9**. Reduction of ketone **5** with NaBH_4_ afforded the C-5 epimeric alcohols **10** and **11** [28]. Given the stereointegrity of the remaining chiral centers during this reduction, their absolute configurations were assigned accordingly. Based on comparative analysis of their NOESY spectra, the more polar epimer was identified as (5*R*)-**10** and the less polar one as (5*S*)-**11**, which was further confirmed by their distinct ^1^H NMR profiles. Acetylation of **10** then furnished derivative **12**. All new compounds were fully characterized by ^1^H NMR, ^13^C NMR, and high-resolution mass spectrometry.

### 2.2. Biological Activity

The in vitro antiproliferative activities of all synthesized compounds against human colorectal carcinoma (HCT116, RKO) and lung carcinoma (A549) cell lines were evaluated using the CCK-8 assay, with doxorubicin (DOX) as a positive control. The results, expressed as IC_50_ values, are summarized in Table 1.

### 2.3. Phenotypic Mechanistic Studies of Compound **8**

**Figure 2:**
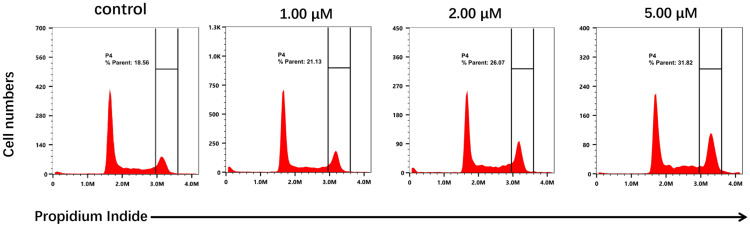
Flow cytometric analyses of the cell cycle distribution of HCT116 cells following treatment with compound **8** (1.00, 2.00 and 5.00 µM) for 24 h and no treatment as a reference control.

Given its activity, the phenotypic mechanism of compound **8** was preliminarily investigated in HCT116 cells. Flow cytometric analysis after PI staining was performed to assess the effect of compound **8** on the cell cycle progression of HCT116 cells. Treatment with **8** at 1.00, 2.00, and 5.00 µM for 24 h induced a concentration-dependent accumulation of cells in the G2/M phase (Figure 2). The percentage of cells in the G2/M phase increased from 18.56% in the untreated control to 21.13%, 26.07%, and 31.82% upon treatment with 1.00, 2.00, and 5.00 µM of **8**, respectively. These results clearly indicate that compound **8** induces G2/M phase cell cycle arrest in a dose-dependent manner.

**Figure 3:**
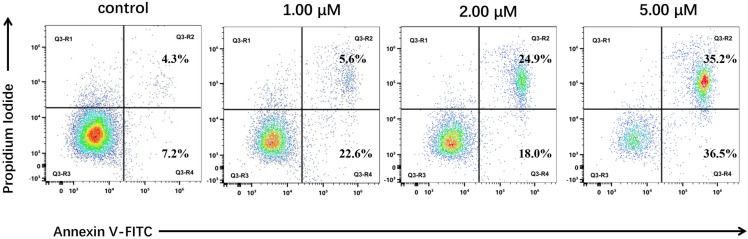
Apoptosis induction in HCT116 cancer cells following treatment with **8** (1.00, 2.00 and 5.00 µM) for 24 h and no treatment (control).

To investigate whether the antiproliferative effect was associated with apoptosis, HCT116 cells were treated with **8** and analyzed by Annexin V-FITC/PI dual staining. The assay demonstrated that compound **8** induced apoptosis in a dose-dependent fashion (Figure 3). After 24 h of treatment, the total apoptotic cell population (early + late apoptosis) increased from 11.5% in the control to 28.2%, 42.9%, and 71.7% at concentrations of 1.00, 2.00, and 5.00 µM, respectively. A detailed analysis revealed a critical transition: at the highest concentration (5.00 µM), a pronounced increase in the late apoptotic cell population (to 35.2%) was observed alongside a significant increase in early apoptosis, resulting in a near 1:1 ratio. This specific distribution at the peak concentration is indicative of an active, asynchronous, and population-wide engagement of the apoptotic cascade. This concentration-dependent progression—from a modest apoptotic response at lower doses to robust, asynchronous apoptosis at the peak dose—provides key mechanistic insight. It supports a sequential model wherein compound **8** first induces a potent G2/M phase arrest, and the sustained failure to resolve this arrest then triggers the canonical apoptotic pathway in a threshold-dependent manner.

## 3. Discussion

### 3.1. Overview: The First Systematic SAR Exploration of the Sarocladione Scaffold

**Figure 4:**
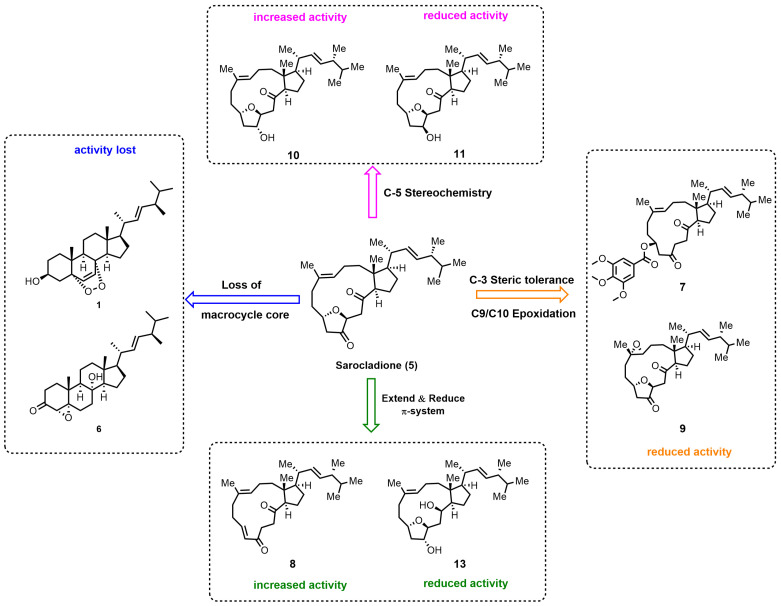
SAR analysis of Sarocladione derivatives.

This study represents the first systematic medicinal chemistry campaign aimed at deciphering the anticancer structure–activity relationships (SAR) of the unique marine-derived sarocladione scaffold. Capitalizing on its recently accomplished biomimetic synthesis [19], we employed a function-oriented design strategy to probe the roles of its defining macrocyclic core, extended conjugation, peripheral substituents, and critical stereochemistry. The resultant SAR transforms sarocladione from a synthetically accessible natural product into a rationalized platform for lead discovery, providing a clear framework for its future optimization. A concise summary of these key structure–activity relationships is presented in Figure 4.

### 3.2. The Macrocyclic Diketone as an Indispensable and Optimizable Core

The complete ablation of activity in analogs lacking the intact 14-membered ring (**1** and **6**) unequivocally establishes the macrocyclic diketone as the fundamental, non-redundant scaffold for bioactivity. This observation aligns with the well-established principle that macrocyclic constraint reduces the entropic penalty of binding by pre-organizing the molecule into its bioactive conformation, a strategy successfully leveraged in many macrocyclic drugs and natural product-derived therapeutics [29]. Within this rigid architecture, strategic modification proved fruitful: extending the π-conjugation (**8**) led to a substantial (~4-fold) potency enhancement, whereas disrupting the conjugated system through reduction (**13**) abolished activity. This underscores that the planar, conjugated diketone is not merely a structural curiosity but a key pharmacophoric element, likely facilitating optimal contact (e.g., via π-stacking or hydrogen bonding) with a putative flat binding pocket.

### 3.3. Exquisite Stereochemical and Steric Demands at Peripheral Sites

Our modifications reveal stringent spatial requirements at the molecular periphery, offering critical insights for future design. The dramatic activity loss upon introducing steric bulk at C-3 (**7**) highlights a sterically sensitive region, suggesting a tightly packed binding environment. Most strikingly, activity is exquisitely dependent on the configuration at the nascent C-5 stereocenter. The (5R)-hydroxy epimer **10** exhibited significantly enhanced potency over both the natural product **5** and its (5S)-counterpart **11**. This profound stereospecificity is a hallmark of high-fidelity molecular recognition in medicinal chemistry, where a single chiral center can dictate binding affinity by orders of magnitude, a phenomenon observed across numerous chiral drugs and bioactive natural products [30]. The subsequent activity drop upon acetylation (**12**) further confirms that the free (5R)-hydroxy group functions as a crucial hydrogen-bond donor/acceptor, forming a specific interaction essential for target engagement.

### 3.4. Selectivity Considerations and Future Perspectives

While this study establishes a robust SAR and identifies a potent lead, profiling against non-malignant cells remains essential to define the therapeutic window and distinguish selective antiproliferation from general cytotoxicity. The differential activity of our compounds across cancer cell lines itself suggests potential for selective action. Future work will therefore prioritize: (1) evaluating lead compounds like **8** against relevant normal cell lines to calculate selectivity indices; (2) in vivo efficacy validation in appropriate animal models; (3) detailed mechanistic studies to identify the precise molecular target(s) upstream of the observed cell cycle arrest; and (4) focused structural optimization based on the established pharmacophore model to further improve potency and drug-like properties.

In summary, the SAR study delineates clear structure–activity trends. Most importantly, these findings indicate that both macrocyclic integrity and conjugated system rigidity are critical determinants of antiproliferative activity. Extension of conjugation and appropriate stereochemical orientation of polar functionalities emerged as two effective strategies for activity enhancement, whereas excessive steric bulk or disruption of the diketone system proved detrimental. These results provide a clear and novel structural framework for anticancer drug discovery.

## 4. Materials and Methods

### 4.1. General Experimental Procedures

All the materials that we used were purchased from suppliers and used without further purification. The structures of target compounds were confirmed by characterization with nuclear magnetic resonance (^1^H-NMR and ^13^C-NMR) and Liquid Chromatography Mass Spectrometry (LC/MS). NMR spectra were recorded on the Bruker AVANCE instrument (Bruker BioSpin GmbH, Rheinstetten, Germany) (600 MHz for ^1^H-NMR and 150 MHz for ^13^C-NMR) at 25 °C, TMS used as the internal standard. Coupling constants were reported in hertz (Hz). Electrospray ionization mass spectrometry (ESI-MS) spectra were recorded on a Q-TOF MicroMass spectrometer (1290-6545 UHPLC-QTOF, MicroMass, Wythenshawe, UK).

Details of the general experimental procedures used are included in the Supporting Information.

### 4.2. Bioactivity Experimental Procedures

#### 4.2.1. Materials and Reagents

The human colorectal carcinoma cell line HCT116, lung carcinoma cell line A549, and colorectal carcinoma cell line RKO were purchased from the Cell Bank of the Chinese Academy of Sciences (Shanghai, China). Dimethyl sulfoxide (DMSO) was purchased from Sigma-Aldrich (St. Louis, MO, USA), and Cell Counting Kit-8 (CCK-8) was purchased from APExBIO (Houston, TX, USA). Dulbecco’s Modified Eagles Medium (DMEM) and trypsin were purchased from Invitrogen (Carlsbad, CA, USA). Fetal bovine serum (FBS) was purchased from Clark Bioscience (Shanghai, China). Annexin V-FITC apoptosis detection kit, cell cycle and apoptosis detection kit, Penicillin-Streptomycin Solution (100×) and Phosphate-Buffered Saline (PBS) were purchased from Beyotime Biotechnology (Shanghai, China).

#### 4.2.2. In Vitro Antiproliferative Activity

The antiproliferative activities were determined using the Cell Counting Kit-8 (CCK-8) assay. Cells within passage 5–20 were used for all experiments. Cells were cultured in Dulbecco’s Modified Eagle Medium (DMEM) supplemented with 10% fetal bovine serum (FBS) and 1% penicillin-streptomycin at 37 °C in a humidified atmosphere containing 5% CO_2_. The cells were seeded into 96-well plates at approximately 9 × 10^3^ cells per well and incubated for 24 h. Subsequently, compounds at different concentrations (0.01, 0.1, 1, 2, 5, 10, 20, 50 μM) were added and incubated with the cells for 24 h. Each well was then treated with CCK-8 solution and incubated at 37 °C for 4 h. Absorbance was measured at a wavelength of 450 nm using a microplate reader. The IC_50_ value was defined as the drug concentration required to inhibit cell proliferation by 50% after 24 h of treatment. The antiproliferative activity (IC_50_) was determined after 24 h of compound treatment. This time point was selected to align with the timeframe for mechanistic studies (cell cycle and apoptosis analysis) and was based on preliminary experiments indicating robust and reproducible cytotoxic effects within this period for this compound series.

#### 4.2.3. Analysis of the Cell Cycle Distribution and Apoptosis by Flow Cytometry

HCT116 cells were inoculated in 12 well plates at a density of 1 × 10^5^ cells per well. After treatment with compound **8** (1 μM, 2 μM and 5 μM) for 24 h, the cells were washed with pre-cooled PBS and collected. The cells were fixed by adding 70% pre-cooled ethanol at 4 °C overnight. The cells were gently washed with pre-cooled PBS, and centrifuge to remove the residual ethanol. Add 100 μL RNaseA (Beyotime) to the cell pellet, gently resuspend the cells, and put them into a 37 °C water bath for 30 min. In the control group, 400 μL propidium iodide (PI, Beyotime) staining solution was added, gently mixed, and incubated in a 4 °C refrigerator for 30 min in the dark. Subsequently, the cycle of stained cells was analyzed by flow cytometry (Cytek Biosciences, Inc., Fremont, CA, USA).

HCT116 cells were inoculated in 12 well plates at a density of 1 × 10^5^ cells per well, and treated with compound **8** (1 μM, 2 μM and 5 μM) for 22 h. Cells were collected, washed twice with pre-cooled PBS, and resuspended by the addition of 400 μL of Binding Buffer according to the manufacturer’s instructions. Then 5 μL of Annexin V-EGFP, 5 μL of propidium iodide (PI) and mixture (Beyotime) were sequentially added and incubated for 20 min in a 5% CO_2_ thermostat incubator protected from light at 37 °C. Stained cells were analyzed for apoptosis by flow cytometry (Cytek^®^ Aurora).

#### 4.2.4. Statistical Analysis

IC_50_ values are expressed as mean ± SD, while flow cytometric data are presented as mean ± SEM. Data analyses were performed via *t*-test for two groups, or via One-way followed by Tukey’s post hoc test for multiple groups using Prism software, version 9.0 (GraphPad, San Diego, CA, USA). Differences were considered statistically significant at *p* < 0.05.

## 5. Conclusions

In conclusion, this work presents a systematic chemical and biological investigation of the marine-derived seco-steroid Sarocladione. Our results establish the essential role of the 14-membered macrocyclic diketone framework and demonstrate that antiproliferative potency can be enhanced through conjugation extension and stereochemical optimization. The most active analog, compound **8**, induces G2/M phase cell cycle arrest followed by apoptosis in HCT116 cells, revealing a clear cell cycle-dependent antiproliferative phenotype. Although selectivity toward non-malignant cells remains to be evaluated, the clear and consistent SAR trends observed here argue against nonspecific cytotoxicity. These findings position Sarocladione as a promising marine natural product-inspired scaffold and provide a solid foundation for future mechanistic and pharmacological studies.

## Figures and Tables

**Table 1:** In vitro antiproliferative activity (IC_50_, µM) of sarocladione derivatives against three human cancer cell lines after 24 h of treatment.

Compounds	IC_50_ (µM) ^1^		
	HCT116	A549	RKO
**1**	>100.00	64.91 ± 3.91	57.14 ± 5.46
**4**	22.86 ± 0.60	13.12 ± 0.21	16.63 ± 0.71
**5** (sarocladione)	7.29 ± 0.57	12.64 ± 0.59	10.33 ± 0.49
**6**	>100.00	41.03 ± 0.88	36.47 ± 1.16
**7**	28.15 ± 1.20	37.44 ± 2.00	25.45 ± 1.11
**8**	1.86 ± 0.31	4.05 ± 0.24	4.68 ± 0.17
**9**	9.97 ± 1.03	27.31 ± 2.15	28.28 ± 1.13
**10**	5.13 ± 0.24	6.25 ± 0.14	5.98 ± 0.19
**11**	13.13 ± 0.16	13.57 ± 0.59	11.27 ± 0.42
**12**	10.22 ± 0.64	14.81 ± 0.74	11.03 ± 0.75
**13**	23.41 ± 1.27	16.33 ± 0.21	16.44 ± 0.71
DOX (Doxorubicin)	0.40 ± 0.05	0.05 ± 0.01	0.25 ± 0.03

^1^ IC_50_: 50% inhibitory concentration and the IC_50_ value expressed as the mean ± SD for three determinations (determined by standard CCK-8 assay for HCT116, A549 and RKO).

## Data Availability

The data presented in this study are available in the article and its Supplementary Information.

## Supplementary Materials

The following supporting information can be downloaded at: https://www.mdpi.com/article/10.3390/md24010048/s1. Experimental procedures: Synthesis of compound **1**−**13**; ^1^H NMR, ^13^C NMR, HR-MS and Single-crystal X-ray diffraction (SCXRD) data of Compounds: Figure S1. ^1^H NMR spectrum of **6** (600 MHz, Chloroform-*d*); Figure S2. ^13^C NMR spectrum of **6** (150 MHz, Chloroform-*d*); Figure S3. HR-ESIMS spectrum of **6**; Figure S4. ^1^H NMR spectrum of **7** (600 MHz, Chloroform-*d*); Figure S5. ^13^C NMR spectrum of **7** (150 MHz, Chloroform-*d*); Figure S6. HR-ESIMS spectrum of **7**; Figure S7. ^1^H NMR spectrum of **8** (600 MHz, Chloroform-*d*); Figure S8. ^13^C NMR spectrum of **8** (150 MHz, Chloroform-*d*); Figure S9. HR-ESIMS spectrum of **8**; Figure S10. ^1^H NMR spectrum of **9** (600 MHz, Chloroform-*d*); Figure S11. ^13^C NMR spectrum of **9** (150 MHz, Chloroform-*d*); Figure S12. NOESY spectrum of **9** (600 MHz,600 MHz, Chloroform-*d*); Figure S13. HR-ESIMS spectrum of **9**; Figure S14. ^1^H NMR spectrum of **10** (600 MHz, Chloroform-*d*); Figure S15. ^13^C NMR spectrum of **10** (150 MHz, Chloroform-*d*); Figure S16. HSQC spectrum of **10** (600 MHz,150 MHz, Chloroform-*d*); Figure S17. HMBC spectrum of **10** (600 MHz,150 MHz, Chloroform-*d*); Figure S18. NOESY spectrum of **10** (600 MHz, 600MHz, Chloroform-*d*); Figure S19. HR-ESIMS spectrum of **10**; Figure S20. ^1^H NMR spectrum of **11** (600 MHz, Chloroform-*d*); Figure S21. ^13^C NMR spectrum of **11** (150 MHz, Chloroform-*d*); Figure S22. HR-ESIMS spectrum of **11**; Figure S23. ^1^H NMR spectrum of **12** (600 MHz, Chloroform-*d*); Figure S24. ^13^C NMR spectrum of **12** (150 MHz, Chloroform-*d*); Figure S25. HR-ESIMS spectrum of **12**; Figure S26. ^1^H NMR spectrum of **13** (600 MHz, Chloroform-*d*); Figure S27. ^13^C NMR spectrum of **13** (150 MHz, Chloroform-*d*); Figure S28. HR-ESIMS spectrum of **13**; Figure S29. Single-crystal X-ray diffraction (SCXRD) data for **13**.

## Abbreviations

The following abbreviations are used in this manuscript:

A549	Human lung adenocarcinoma cell line
Ac2O	Acetic anhydride
Boc2O	Di-tert-butyl dicarbonate
CCK-8	Cell Counting Kit-8
DMAP	4-Dimethylaminopyridine
DMEM	Dulbecco’s Modified Eagle Medium
DMSO	Dimethyl sulfoxide
Et3N	Triethylamine
FBS	Fetal bovine serum
G2/M	Gap 2/Mitosis phase
HCT116	Human colorectal carcinoma cell line
IC50	Half maximal inhibitory concentration
Im	Imidazole
m-CPBA	meta-Chloroperoxybenzoic acid
MeOH	Methanol
PADA	Potassium (E)-diazene-1,2-dicarboxylate
PBS	Phosphate-buffered saline
PCC	Pyridinium chlorochromate
PI	Propidium iodide
Py	Pyridine
RKO	Human colorectal carcinoma cell line
SAR	Structure–activity relationship
TESCl	Chlorotriethylsilane
