# Supplementary material for: Design and Synthesis of Marine Sarocladione Derivatives with Potential Anticancer Activity
Source: Mar Drugs. 2026 Jan 20;24(1):48. doi: 10.3390/md24010048 (PMC12843125; doi:10.3390/md24010048)
Supplement: Supplementary file 1 [file marinedrugs-24-00048-s001.zip › marinedrugs-4095346-supplementary.pdf]

Supporting Information

Design and Synthesis of Marine Sarocladione Derivatives with Potential Anticancer Activity

Table of Contents

1. **Experimental procedures** 3

2. **^{1}H NMR, ^{13}C NMR, HR-MS and Single-crystal X-ray diffraction (SCXRD) data of Compounds** 10

1. Experimental procedures

Synthesis of compound 1

Ergosterol (500 mg, 1.26 mmol) and tetraphenylporphyrin (TPP) (7.75 mg, 0.01 mmol) were dissolved in dichloromethane (25 mL). The solution was stirred and oxygen was bubbled through it at 0 °C while being irradiated with an incandescent lamp for 1 h. The reaction was monitored by TLC. Upon completion, the light source was removed and the mixture was concentrated under reduced pressure. The residue was purified by silica gel column chromatography (petroleum ether/ethyl acetate = 4:1 to 3:1) to afford the compound **1** (white solid, 355 mg, 66%) [19]. ^{1}H NMR (400 MHz, Chloroform-*d*) δ 6.50 (d, *J* = 8.5 Hz, 1H), 6.24 (d, *J* = 8.5 Hz, 1H), 5.22 (dd, *J* = 15.3, 7.4 Hz, 1H), 5.14 (dd, *J* = 15.2, 8.1 Hz, 1H), 3.97 (m, 1H), 2.11 (m, 1H), 2.06 – 1.89 (m, 4H), 1.89 – 1.81 (m, 2H), 1.75 (m, 1H), 1.69 (m, 1H), 1.65 – 1.56 (m, 2H), 1.50 (m, 4H), 1.46 – 1.41 (m, 1H), 1.38 (m, 1H), 1.35 – 1.27 (m, 1H), 1.23 (dd, *J* = 9.8, 6.6 Hz, 3H), 1.00 (d, *J* = 6.5 Hz, 3H), 0.93 – 0.87 (m, 6H), 0.85 – 0.80 (m, 9H).

Synthesis of compound 2

The preparation of PADA

To a cooled (0 °C) 40% aq. KOH solution (18.6 ml) was added azodicarbonamide (3.0 g, 25.8 mmol) in small portions over a period of 2 h. The temperature was always kept at 0 °C during each addition. After stirring for additional two hours, the bright yellow solid product was filtered off using a Büchner funnel, and the solid was washed 10 times with 2 ml of precooled (0 °C) MeOH (Note: Part of the product was precipitated from the filtrate and was isolated by re-filtration). Drying under high vacuum gave the title compound PADA (4.8 g, 96%) as a yellow solid.

Compound **1** (2.2 g, 5.2 mmol) was added to a 250 mL dry single-necked flask, followed by the addition of anhydrous MeOH (70 mL) and anhydrous DCM (35 mL). After the substrate dissolved, PADA (4.1 g, 21.2 mmol) was added. The mixture was cooled to 0°C, and a solution of AcOH in MeOH (2.4 mL AcOH in 4.2 mL MeOH, 42.4 mmol) was slowly added dropwise at 0°C. After 30 minutes of addition, the system was stirred at room temperature overnight. A small amount of the solution was filtered through a syringe filter, and the solvent was removed under reduced pressure for NMR analysis, confirming complete consumption of the starting material. Crude silica gel (100-200 mesh) was directly added, and the solvent was removed under reduced pressure. Column chromatography (SiO2, petroleum ether/ethyl acetate = 3:1 to 2:1) was performed to isolate compound **2** (white solid, 2.2 g, 99%) [19]. ^{1}H NMR (400 MHz, Chloroform-*d*) δ 5.20 (dd, J = 15.3, 7.4 Hz,1H), 5.12 (dd, J = 15.3, 8.1 Hz, 1H), 3.83 – 3.73 (m, 1H), 2.02 – 1.90 (m, 3H), 1.88 – 1.82 (m, 2H), 1.82 – 1.73 (m, 4H), 1.71 – 1.61 (m, 2H), 1.60 – 1.49 (m, 4H), 1.49 – 1.42 (m, 3H), 1.41 – 1.29 (m, 2H),1.28 – 1.18 (m, 3H),

1.18 – 1.08 (m, 1H), 0.99 (s, 3H), 0.98 (d, J = 6.6 Hz, 3H), 0.90 (d, J = 6.8 Hz, 3H), 0.82 (d, J = 6.7 Hz, 3H), 0.80 (d, J = 6.7 Hz, 3H), 0.72 (s, 3H).

Synthesis of compound 3

Compound **2** (2 g, 4.6 mmol) was added to a 250 mL dry single-necked flask, followed by the addition of anhydrous DCM (93 mL), DMAP (170 mg, 1.38 mmol), imidazole (1.3 g, 18.4 mmol), and TESCl (1.60 mL, 9.2 mmol). The reaction was stirred at room temperature for 1 hour, and the completion was monitored by TLC. The reaction was quenched by adding a saturated $NaHCO_3$ solution. The aqueous layer was extracted with DCM (150 mL × 3), and the combined organic phases were dried over anhydrous sodium sulfate, filtered, and concentrated under reduced pressure. Purification by column chromatography (SiO_2, petroleum ether/ethyl acetate = 80:1 to 60:1) afforded compound **3** (white solid, 2.4 g, 95%) [19]. ^{1}H NMR (400 MHz, Chloroform-*d*) δ 5.20 (dd, *J* = 15.3, 7.3 Hz, 1H), 5.11 (dd, *J* = 15.3, 8.2 Hz, 1H), 3.72 (m, 1H), 2.02 – 1.90 (m, 3H), 1.84 (dd, *J* = 13.4, 7.4 Hz, 2H), 1.79 – 1.72 (m, 3H), 1.71 – 1.61 (m, 3H), 1.61 – 1.45 (m, 8H), 1.41 – 1.29 (m, 2H), 1.29 – 1.17 (m, 3H), 1.12 (m, 1H), 0.98 (s, 3H), 0.97 (d, J = 6.6 Hz, 3H), 0.92 (t, J = 7.9 Hz, 9H), 0.89 (d, J = 6.8 Hz, 3H), 0.82 (d, J = 6.7 Hz, 3H), 0.80 (d, J = 6.7 Hz, 3H), 0.71 (s, 3H), 0.56 (q, *J* = 7.9 Hz, 6H).

Synthesis of compound 4

Compound 3 (50 mg, 0.0918 mmol) was added to a dry 25 mL Schlenk tube, followed by the addition of $[RuCl_2(CO)_3]_2$ (2.4 mg, 0.00459 mmol). The tube was evacuated and backfilled with Argon three times. Anhydrous toluene (2 mL) was added, and the mixture was stirred to dissolve the solids. A dry 10 mL microwave tube equipped with a dedicated stir bar was evacuated and backfilled with Argon three times. The substrate solution in toluene was transferred to the microwave tube, followed by rinsing with a small amount of toluene (1.7 mL). The reaction mixture was heated to 150 °C using microwave irradiation for 20 minutes. The reaction was then stopped and cooled to room temperature. THF (5 mL), pyridine (1.5 mL), and HF•pyridine (0.5 mL) were sequentially added, and the mixture was stirred at room temperature for 15 minutes. The reaction was quenched by adding a saturated $NaHCO_3$ solution. The aqueous layer was extracted with EtOAc (50 mL × 3), and the combined organic phases were dried over anhydrous sodium sulfate, filtered, and concentrated under reduced pressure. Purification by column chromatography (SiO_2, petroleum ether/ethyl acetate = 5:1 to 4:1 to 3:1) afforded compound **4** (colorless oil, 28.2 mg, 71%)[19]. ^{1}H NMR (600 MHz, Chloroform-*d*) δ 5.24 – 5.19 (m, 2H), 5.17 (dd, *J* = 8.8, 3.7 Hz, 1H), 3.76 (m, 1H), 3.11 – 2.97 (m, 1H), 2.89 (dd, *J* = 15.6, 2.6 Hz, 1H), 2.81 – 2.68 (m, 3H), 2.51 (m, 1H), 2.46 – 2.40 (m, 1H), 2.16 (m, 6H), 2.08 – 1.98 (m,

1H), 1.88 (m, 5H), 1.76 – 1.66 (m, 3H), 1.63 (d, *J* = 5.9 Hz, 1H), 1.56 (m, 3H), 1.50 – 1.42 (m, 3H), 1.31 (m, 1H), 1.03 (d, *J* = 6.8 Hz, 3H), 0.91 (d, *J* = 6.9 Hz, 3H), 0.82 (m, 6H).

Synthesis of compound 5

Condition A

Compound **1** (53 mg, 0.124 mmol) was added to a dry 50 mL single-necked flask equipped with a condenser. The flask was evacuated and backfilled with argon (three cycles). A mixture of AcOH/H_2O (12.5 mL, 100:1 v/v) was added, and the reaction mixture was stirred until complete dissolution was achieved. It was then heated to 140°C to reflux for 4 hours. After completion, the reaction was stopped and cooled to room temperature. The solvent was removed under reduced pressure. The crude product was purified by column chromatography (SiO_2, petroleum ether/ethyl acetate = 12:1 to 10:1) to afford compound **5** (colorless oil, 8 mg, 15% yield). ^{1}H NMR (600 MHz, Chloroform-*d*) δ 5.49 (t, *J* = 8.2 Hz, 1H), 5.23 (dd, J = 15.5, 7.5 Hz, 1H), 5.20 (dd, J = 15.5, 6.0 Hz, 1H), 4.12 (tdd, J = 11.3, 5.0, 1.6 Hz, 1H), 4.06 (dd, *J* = 5.6, 3.8 Hz, 1H), 2.95 (dd, *J* = 14.7, 5.5 Hz, 1H), 2.83 (dd, *J* = 10.9, 9.5 Hz, 1H), 2.64 (dd, *J* = 14.8, 3.9 Hz, 1H), 2.56 (dd, *J* = 17.0, 5.2 Hz, 1H), 2.33 (m, 1H), 2.30 – 2.24 (m, 1H), 2.20 – 2.16 (m, 1H), 2.14 – 2.10 (m, 1H), 2.06 – 1.94 (m, 3H), 1.90 – 1.82 (m, 3H), 1.81 – 1.74 (m, 3H), 1.64 – 1.59 (m, 2H), 1.57 (d, *J* = 1.3 Hz, 3H), 1.49 – 1.44 (m, 1H), 1.40 (m, 1H), 1.01 (d, *J* = 6.7 Hz, 3H), 0.91 (d, *J* = 6.9 Hz, 3H), 0.88 (s, 3H), 0.84 – 0.81 (m, 6H).

Condition B

Compound **1** (50 mg, 0.117 mmol) was added to a dry 25 mL Schlenk tube. $[RuCl_2(CO)_3]_2$ (3.0 mg, 0.00585 mmol) was added, and the tube was evacuated and backfilled with Argon three times. Anhydrous toluene (2.7 mL) was added, and the mixture was stirred to dissolve the solids. A dry 10 mL microwave tube equipped with a dedicated stir bar was evacuated and backfilled with Argon three times. The substrate solution in toluene was transferred to the microwave tube, and the Schlenk tube was rinsed with a small amount of anhydrous toluene (2.0 mL), which was also transferred to the microwave tube. The reaction mixture was heated to 150 °C using microwave irradiation for 20 minutes. After the reaction was complete, it was cooled to room temperature, and the solvent was removed under reduced pressure. Purification by column chromatography (SiO_2, petroleum ether/ethyl acetate = 12:1 to 10:1) afforded compound **5** (colorless oil, 10 mg, 20% yield) [19].

Synthesis of compound 6

Compound 2 (50 mg, 0.116 mmol) was dissolved in 0.4 mL of acetone. Jones reagent (17.4 mg of chromium trioxide dissolved in 0.14 mL of water and 0.016 mL of sulfuric acid) was added dropwise. The reaction was stirred for 1 hour, then quenched with methanol. The mixture was extracted with

ethyl acetate (three times), and the combined organic layers were washed with saturated sodium chloride solution. Purification by silica gel column chromatography (SiO_2, hexanes/ ethyl acetate = 9:1 to 5:1) afforded compound 6 (white solid,12 mg, 23% yield). $[\alpha]_D^{20}$ −6.5 (*c* 0.1, MeOH); ^{1}H NMR (600 MHz, Chloroform-*d*) δ 5.21 (dd, *J* = 15.3, 7.6 Hz, 1H), 5.15 (dd, *J* = 15.3, 8.2 Hz, 1H), 3.23 (s, 1H), 2.54 (s, 1H), 2.42 – 2.36 (m, 2H), 2.30 – 2.23 (m, 1H), 2.17 – 2.11 (m, 1H), 2.02 – 1.97 (m, 1H), 1.95 (m, 1H), 1.87 – 1.82 (m, 3H), 1.72 (m, 2H), 1.64 – 1.59 (m, 3H), 1.50 – 1.45 (m, 3H), 1.40 – 1.36 (m, 1H), 1.28 (m, 1H), 1.24 – 1.15 (m, 3H), 1.06 (s, 3H), 1.00 (d, *J* = 6.6 Hz, 3H), 0.91 (d, *J* = 6.9 Hz, 3H), 0.83 (m, 6H), 0.77 (s, 3H). ^{13}C NMR (150 MHz, $CDCl_3$) δ 207.10, 135.43, 132.34, 74.49, 69.51, 63.32, 59.85, 57.12, 53.86, 42.96, 42.88, 40.17, 39.54, 35.94, 33.39, 33.23, 32.74, 28.02, 25.68, 24.59, 24.48, 20.84, 20.46, 20.19, 20.11, 19.78, 17.79, 12.79. HR-ESI-MS: $[M+H]^+$ calcd for $C_{28}H_{45}O_3$ 429.3363, found: 429.3359.

Synthesis of compound 7

To a solution of compound **4** (28 mg, 0.07 mmol) and DMAP (3.2 mg, 0.03 mmol) in dry pyridine (2 mL) was added 3,4,5-trimethoxybenzoyl chloride (60.2 mg, 0.26 mmol) and Et_3N (52.8 mg, 0.52 mmol). The reaction mixture was stirred at room temperature for 2 h. Upon completion, the mixture was diluted with water and extracted with EtOAc (3 × 10 mL). The combined organic layers were washed with dilute HCl and brine, dried over Na_2SO_4, and concentrated under reduced pressure. The residue was purified by column chromatography on silica gel (petroleum ether/ethyl acetate = 9:1 to 8:1) to afford compound **7** (colorless oil, 28 mg, 68%). $[\alpha]_D^{20}$ −256 (*c* 0.1, MeOH); ^{1}H NMR (600 MHz, Chloroform-*d*) δ 7.27 (s, 2H), 5.31 – 5.28 (m, 1H), 5.22 (m, 3H), 3.91 (s, 6H), 3.90 (s, 3H), 2.95 (m, 2H), 2.83 (m, 1H), 2.71 – 2.61 (m, 3H), 2.47 (m, 1H), 2.29 – 2.04 (m, 7H), 2.01 – 1.95 (m, 1H), 1.90 – 1.83 (m, 2H), 1.82 – 1.73 (m, 4H), 1.65 – 1.57 (m, 3H), 1.51 – 1.44 (m, 2H), 1.05 (d, *J* = 6.8 Hz, 3H), 0.92 (d, *J* = 6.8 Hz, 3H), 0.83 (m, 6H), 0.79 (s, 3H). ^{13}C NMR (150 MHz, $CDCl_3$) δ 210.35, 207.39, 165.88, 153.09, 142.54, 134.63, 133.79, 132.98, 128.47, 125.17, 107.01, 70.57, 61.05, 57.52, 56.39, 50.17, 47.98, 45.87, 43.08, 39.11, 38.13, 37.95, 37.41, 34.57, 33.21, 30.27, 26.27, 23.58, 21.62, 21.45, 20.13, 19.81, 17.75, 17.67, 15.93. HR-ESI-MS: $[M+Na]^+$ calcd for $C_{38}H_{56}O_7Na$ 647.3918, found: 647.3914.

Synthesis of compound 8

To a stirred solution of compound **4** (60 mg, 0.14 mmol) in DMA was added aminomethylsulfonyl chloride (32.2 mg, 0.28 mmol) at 0 °C. The mixture was stirred for 3 h at this temperature. After

completion, the mixture was diluted with ethyl acetate and washed with water several times. The organic layer was dried over anhydrous Na_2SO_4 and concentrated under reduced pressure. The residue was purified by column chromatography on silica gel (petroleum ether/ethyl acetate = 15:1 to 9:1) to afford compound **8** (colorless oil, 30 mg, 43%).$[\alpha]_D^{20}$ −121.2 (*c* 0.1, MeOH); ^{1}H NMR (600 MHz, Chloroform-*d*) δ 6.57 – 6.50 (m, 1H), 5.92 (d, *J* = 16.1 Hz, 1H), 5.24 – 5.16 (m, 3H), 2.89 (m, 1H), 2.82 (dd, *J* = 10.6, 8.6 Hz, 1H), 2.67 (m , 1H), 2.55 – 2.47 (m, 2H), 2.44 – 2.18 (m, 5H), 2.14 (m, 2H), 2.10 – 2.04 (m, 1H), 1.91 (dd, *J* = 15.2, 7.7 Hz, 1H), 1.85 (td, *J* = 12.4, 11.2, 4.6 Hz, 2H), 1.66 (m, 3H), 1.56 (t, *J* = 7.9 Hz, 1H), 1.45 (m, 3H), 1.03 (d, *J* = 6.8 Hz, 3H), 0.90 (d, *J* = 6.8 Hz, 3H), 0.82 (m, 6H), 0.78 (s, 3H). ^{13}C NMR (150 MHz, $CDCl_3$) δ 209.82, 201.51, 147.82, 134.68, 132.94, 132.92, 130.27, 128.43, 58.05, 50.50, 48.32, 43.07, 41.79, 38.65, 38.32, 37.98, 34.76, 33.21, 29.45, 26.71, 23.88, 21.62, 21.42, 20.13, 19.81, 17.77, 17.68, 15.90. HR-ESI-MS: $[M+H]^+$ calcd for $C_{28}H_{45}O_2$ 413.3414, found: 413.3414.

Synthesis of compound 9

Compound **5** (12 mg, 0.03 mmol) was dissolved in dichloromethane (0.6 mL), and sodium bicarbonate (11.8 mg, 0.14 mmol) was added. The mixture was cooled to 0 °C. m-CPBA (85%, 10.0 mg, 0.04 mmol) was added, and the reaction was stirred at 0 °C. After the reaction was complete, the mixture was quenched with saturated sodium sulfite solution and extracted with dichloromethane. The solvent was removed under reduced pressure. Purification by column chromatography (petroleum ether/ethyl acetate = 4:1) afforded compound **9** (colorless oil, 6.4 mg, 51%).$[\alpha]_D^{20}$ 21.9 (*c* 0.7, MeOH); ^{1}H NMR (600 MHz, Chloroform-*d*) δ 5.20 (dd, *J* = 4.9, 2.1 Hz, 2H), 4.21 (m, 1H), 4.05 (d, *J* = 5.8 Hz, 1H), 3.06 (dd, *J* = 15.3, 6.0 Hz, 1H), 2.90 (t, *J* = 6.6 Hz, 1H), 2.81 – 2.72 (m, 3H), 2.27 – 2.22 (m, 1H), 2.08 (m, 3H), 2.00 – 1.95 (m, 2H), 1.82 (m, 3H), 1.67 – 1.56 (m, 5H), 1.49 – 1.42 (m, 3H), 1.22 (s, 3H), 0.99 (d, *J* = 6.8 Hz, 6H), 0.91 (d, *J* = 6.8 Hz, 3H), 0.82 (m, 6H). ^{13}C NMR (150 MHz, $CDCl_3$) δ 214.85, 211.54, 134.63, 133.03, 77.29, 75.02, 63.84, 61.65, 59.81, 45.72, 45.65, 43.56, 43.07, 37.75, 34.57, 33.21, 31.86, 26.14, 23.92, 23.81, 22.21, 21.48, 20.13, 19.81, 17.72, 15.49. HR-ESI-MS: $[M+H]^+$ calcd for $C_{28}H_{45}O_4$ 445.3312, found: 445.3312.

Synthesis of compound 10 and 11

Sodium borohydride (1.8 mg, 0.048 mmol) was dissolved in anhydrous tetrahydrofuran (10 mL) to prepare a stock solution. An aliquot (1.5 mL, containing ~0.27 mg $NaBH_4$, 0.007 mmol) of this solution was transferred to a solution of compound 5 (6 mg, 0.014 mmol) in THF. The reaction was stirred for 1 hour, then quenched with water and concentrated under reduced pressure. The mixture was extracted

with ethyl acetate. The organic phase was washed with saturated sodium chloride solution, concentrated under reduced pressure, and purified by column chromatography (petroleum ether/ethyl acetate = 3:1 to 2:1) to afford compound **10** (white solid, 3.3 mg, 55%) and compound **11** (white solid, 1.2 mg, 20%).

Compound **10**:

$[\alpha]_D^{20}$ −46.5 (*c* 0.1, MeOH); ^{1}H NMR (600 MHz, Chloroform-*d*) δ 5.29 (t, *J* = 7.3 Hz, 1H), 5.24 (dd, *J* = 15.3, 7.9 Hz, 1H), 5.19 (dd, *J* = 15.2, 7.1 Hz, 1H), 4.30 (m, 1H), 3.95 (m, 1H), 3.82 (m, 1H), 3.09 (t, *J* = 9.4 Hz, 1H), 2.67 (dd, *J* = 13.1, 3.8 Hz, 1H), 2.36 – 2.28 (m, 2H), 2.24 – 1.96 (m, 6H), 1.88 – 1.83 (m, 3H), 1.76 – 1.61 (m, 6H), 1.59 (s, 3H), 1.51 – 1.45 (m, 2H), 1.43 – 1.38 (m, 1H), 1.02 (d, *J* = 6.8 Hz, 3H), 0.91 (d, *J* = 6.8 Hz, 3H), 0.85 (s, 3H), 0.82 (m, 6H). ^{13}C NMR (150 MHz, $CDCl_3$) δ 211.94, 135.03, 132.82, 132.68, 128.27, 82.26, 77.10, 76.79, 56.86, 49.94, 46.48, 46.23, 43.09, 41.99, 37.68, 37.60, 37.53, 33.25, 31.44, 26.93, 25.08, 22.69, 21.80, 20.15, 19.82, 18.32, 17.70, 15.33. HR-ESI-MS: $[M+H-H_2O]^+$ calcd for $C_{28}H_{45}O_2$ 413.3415, found: 413.3405.

Compound **11**:

$[\alpha]_D^{20}$ −17.7 (*c* 0.7, MeOH); ^{1}H NMR (600 MHz, Chloroform-*d*) δ 5.39 (t, *J* = 7.5 Hz, 1H), 5.22 (m, 2H), 4.96 (d, *J* = 9.0 Hz, 1H), 4.34 – 4.30 (m, 2H), 3.76 (m, 1H), 3.09 (dd, *J* = 14.1, 6.2 Hz, 1H), 2.95 (t, *J* = 10.4 Hz, 1H), 2.49 (dd, *J* = 14.1, 2.4 Hz, 1H), 2.21 – 2.13 (m, 3H), 2.06 – 1.97 (m, 5H), 1.86 (m, 1H), 1.80 – 1.68 (m, 5H), 1.68 – 1.63 (m, 2H), 1.57 (s, 3H), 1.49 – 1.32 (m, 5H), 1.03 (d, *J* = 6.8 Hz, 3H), 0.92 (d, *J* = 6.8 Hz, 3H), 0.87 (s, 3H), 0.84 – 0.81 (m, 6H). ^{13}C NMR (150 MHz, $CDCl_3$) δ 215.23, 134.83, 133.34, 132.83, 127.91, 80.00, 76.05, 73.92, 59.97, 45.81, 43.72, 43.11, 42.09, 37.54, 37.16, 36.95, 33.25, 31.47, 29.85, 25.59, 24.28, 22.42, 21.74, 20.16, 19.82, 17.71, 16.86, 14.79. HR-ESI-MS: $[M+H]^+$ calcd for $C_{28}H_{47}O_3$ 431.3520, found: 431.3516.

Synthesis of compound 12

Compound **10** (2.8 mg, 6.5 μmol) was dissolved in dichloromethane (120 μL). Pyridine (30 μL) and acetic anhydride (6 μL) were added, and the reaction mixture was stirred for 1 hour. After completion, the reaction was quenched with water and extracted with ethyl acetate (3 times). The combined organic layers were washed with saturated sodium bicarbonate solution and saturated sodium chloride solution, then concentrated under reduced pressure. Purification by column chromatography (petroleum ether/ethyl acetate = 8:1) afforded compound **12** (colorless oil, 2.8 mg, 91%). $[\alpha]_D^{20}$ −42.3 (*c* 0.1, MeOH); ^{1}H NMR (600 MHz, Chloroform-*d*) δ 5.29 (t, *J* = 7.2 Hz, 1H), 5.23 (dd, *J* = 15.2, 7.7 Hz, 1H), 5.19 (dd, *J* = 15.0, 6.8 Hz, 1H), 5.12 (m, 1H), 4.12 (dt, *J* = 8.8, 3.8 Hz, 1H), 3.81 (m, 1H), 3.03 (t, *J* =

9.5 Hz, 1H), 2.70 (dd, *J* = 13.6, 4.0 Hz, 1H), 2.48 – 2.40 (m, 2H), 2.20 (m, 1H), 2.16 – 2.06 (m, 3H), 2.04 (s, 3H), 2.02 (dd, *J* = 7.9, 4.4 Hz, 1H), 1.88 – 1.80 (m, 4H), 1.77 – 1.62 (m, 5H), 1.59 (d, J = 1.3 Hz, 3H), 1.55 – 1.52 (m, 1H), 1.49 – 1.39 (m, 2H), 1.02 (d, *J* = 6.8 Hz, 3H), 0.91 (d, *J* = 6.8 Hz, 3H), 0.85 (s, 3H), 0.82 (m, 6H). ^{13}C NMR (150 MHz, $CDCl_3$) δ 211.48, 171.09, 135.02, 132.72, 132.68, 128.59, 80.67, 78.96, 77.05, 57.21, 49.99, 46.50, 46.18, 43.09, 39.00, 37.84, 37.65, 37.48, 33.25, 30.98, 26.87, 24.81, 22.46, 21.88, 21.18, 20.15, 19.83, 18.23, 17.69, 15.20. HR-ESI-MS: $[M+H]^+$ calcd for $C_{30}H_{49}O_4$ 473.3626, found: 473.3623.

Synthesis of compound 13

At 0 °C, compound 5 (218 mg, 0.5mmol) was dissolved in tetrahydrofuran (15 mL). Sodium borohydride (38.5 mg, 1mmol) was added, and the reaction was stirred overnight. The reaction was quenched with water and concentrated under reduced pressure. The mixture was extracted with ethyl acetate. The organic phase was washed with saturated sodium chloride solution, concentrated under reduced pressure, and purified by column chromatography (petroleum ether/ethyl acetate = 5:1) to afford compound 13 (white solid, 38.9 mg, 18%).$[\alpha]_D^{20}$ −140.6 (*c* 0.3, MeOH); ^{1}H NMR (600 MHz, Chloroform-*d*) δ 5.31 – 5.29 (m, 1H), 5.29 – 5.25 (m, 1H), 5.18 (dd, *J* = 15.3, 7.9 Hz, 1H), 4.21 – 4.15 (m, 1H), 3.91 – 3.86 (m, 1H), 3.69 – 3.60 (m, 2H), 2.44 (m, 1H), 2.33 (dt, *J* = 15.5, 11.0 Hz, 1H), 2.24 – 2.17 (m, 3H), 2.14 – 2.07 (m, 2H), 1.94 – 1.76 (m, 6H), 1.67 – 1.59 (m, 5H), 1.55 – 1.42 (m, 5H), 1.37 (dd, *J* = 12.2, 6.2 Hz, 1H), 1.05 (d, *J* = 6.9 Hz, 3H), 1.00 – 0.96 (m, 1H), 0.92 (d, *J* = 6.9 Hz, 3H), 0.83 (m, 6H), 0.81 (s, 3H).^{13}C NMR (150 MHz, $CDCl_3$) δ 135.10, 132.81, 132.49, 128.97, 80.08, 79.10, 77.75, 73.61, 49.88, 47.07, 45.50, 43.21, 40.88, 37.81, 37.01, 36.85, 36.23, 33.29, 31.30, 25.33, 25.30, 22.93, 22.54, 20.19, 19.83, 17.76, 17.11, 16.35. HR-ESI-MS: $[M+H]^+$ calcd for $C_{28}H_{49}O_3$ 433.3676, found: 433.3665.

2. ^{1}H NMR, ^{13}C NMR, HR-MS and Single-crystal X-ray diffraction (SCXRD) data of Compounds

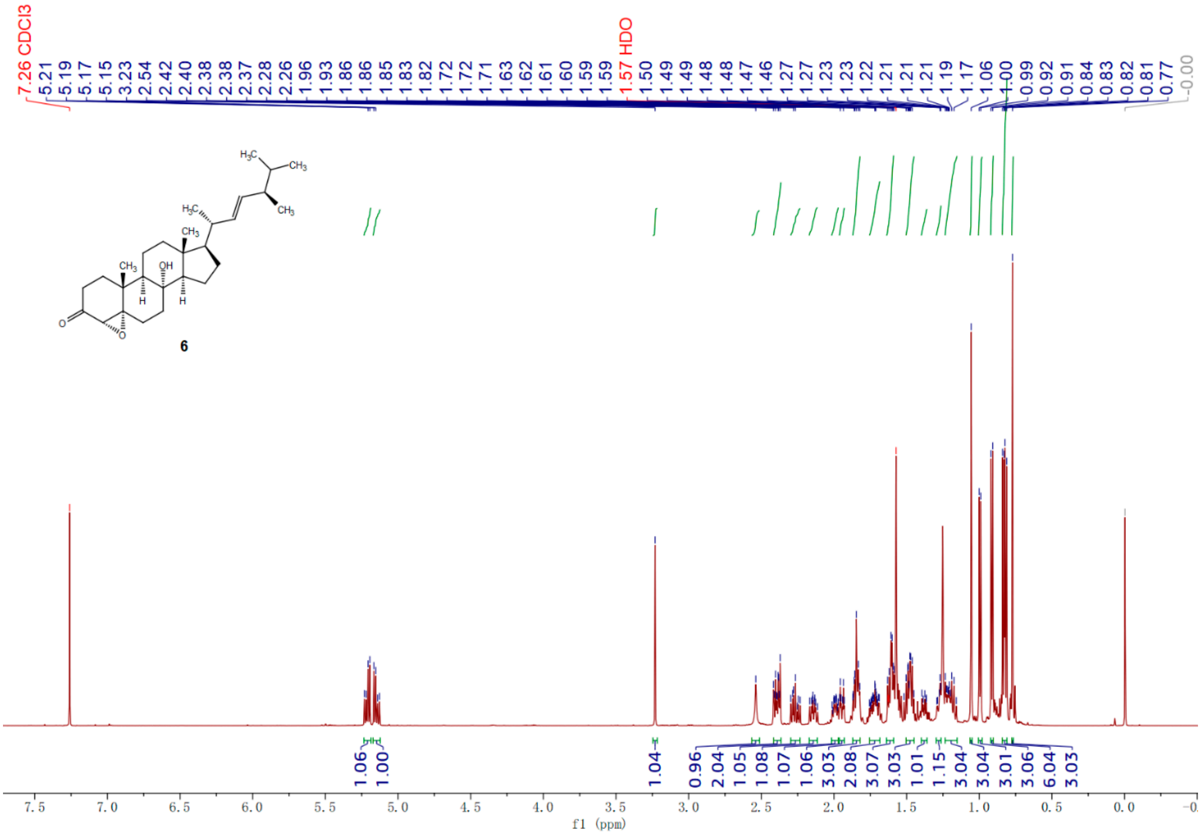

Figure S1. ^{1}H NMR spectrum of 6 (600 MHz, Chloroform-*d*)

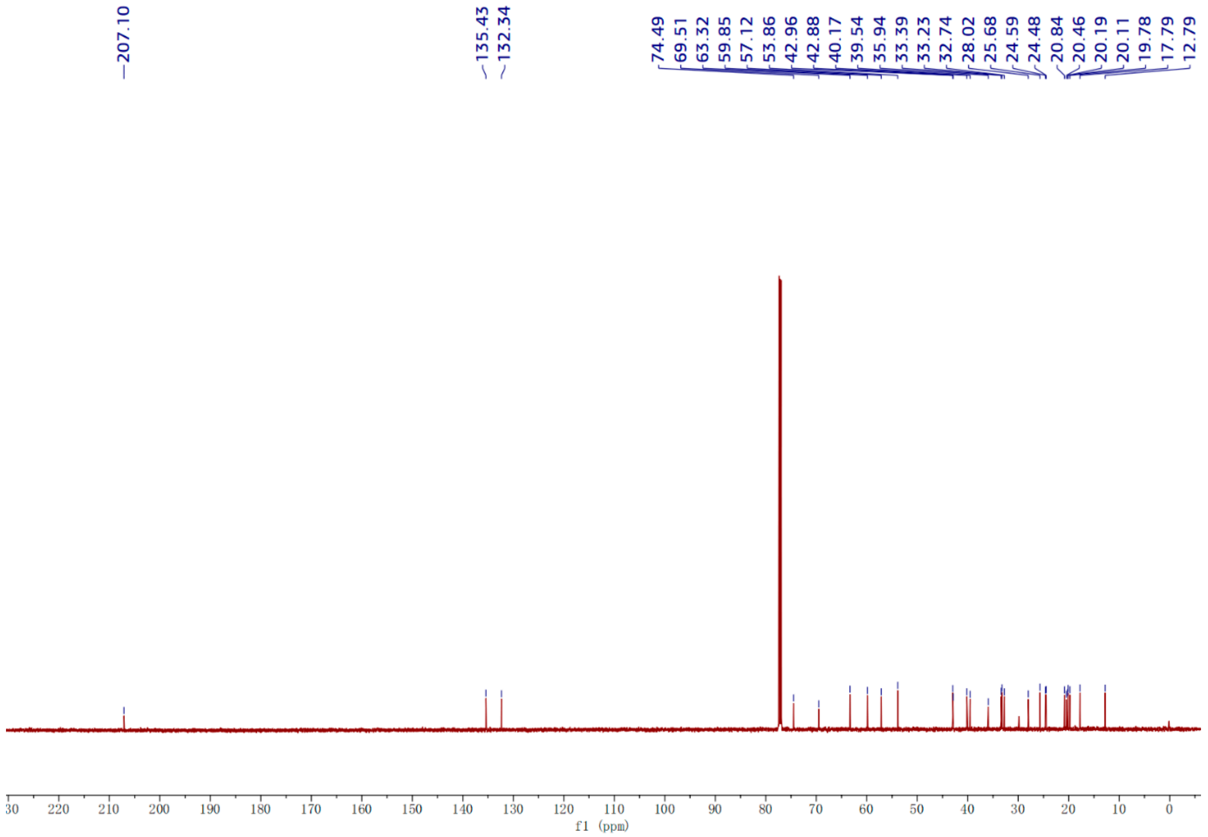

Figure S2. ^{13}C NMR spectrum of 6 (150 MHz, Chloroform-*d*)

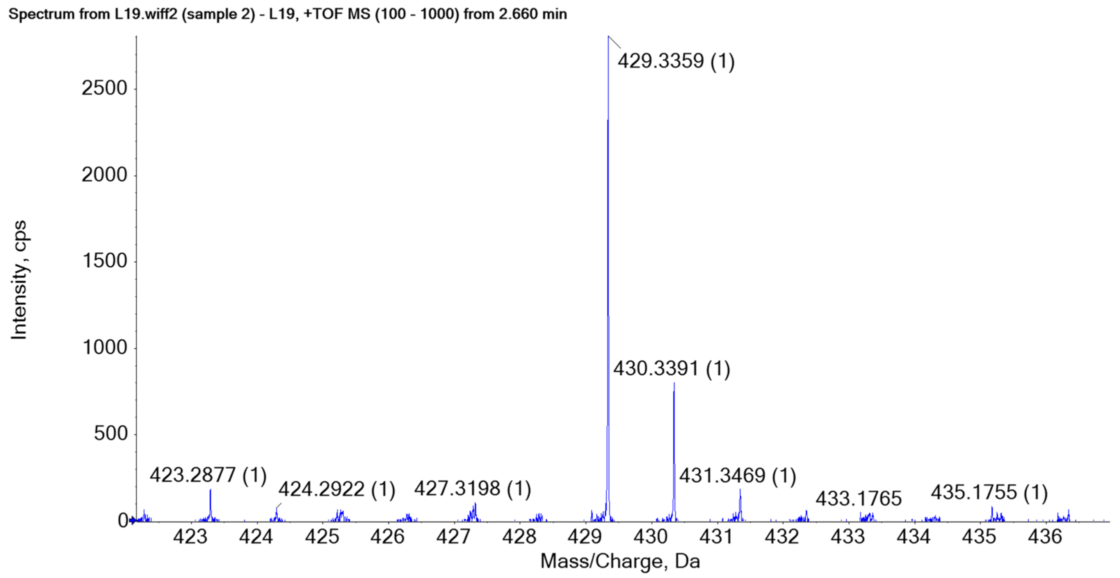

Formula Calculator Results

Measured m/z	Cal m/z	Error(mmu)	Error(ppm)	Ion Formula	Ion
429.3359	429.3363	-0.4	-1.0	C28H45O3	[M+H]+

Figure S3. HR-ESIMS spectrum of 6

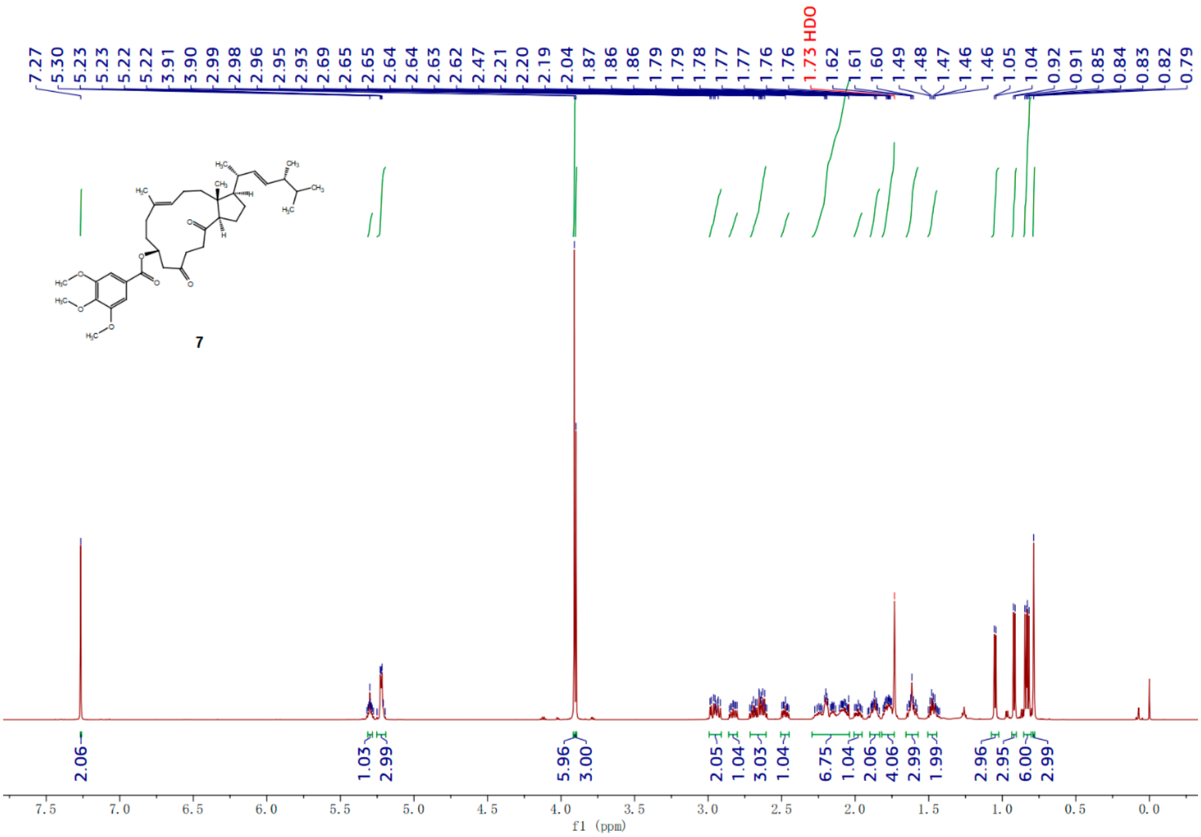

Figure S4. ^{1}H NMR spectrum of 7 (600 MHz, Chloroform-*d*)

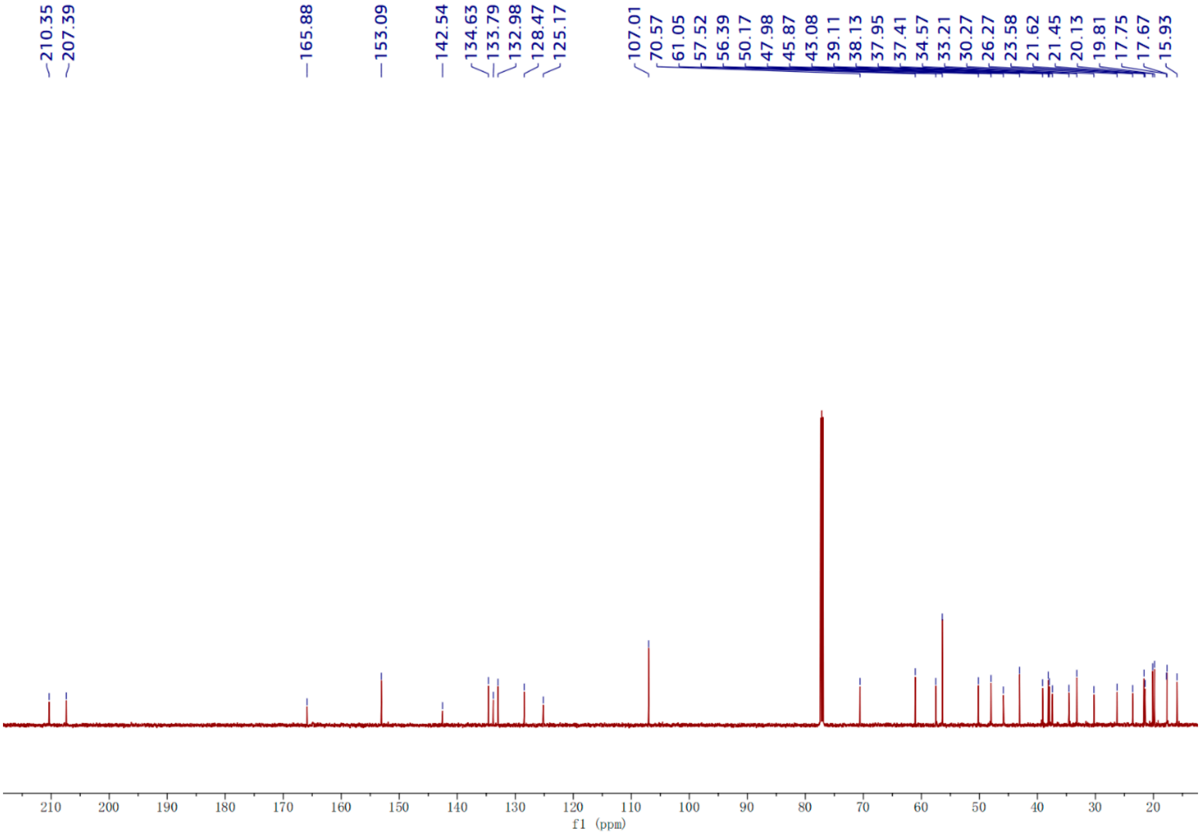

Figure S5. ^{13}C NMR spectrum of 7 (150 MHz, Chloroform-*d*)

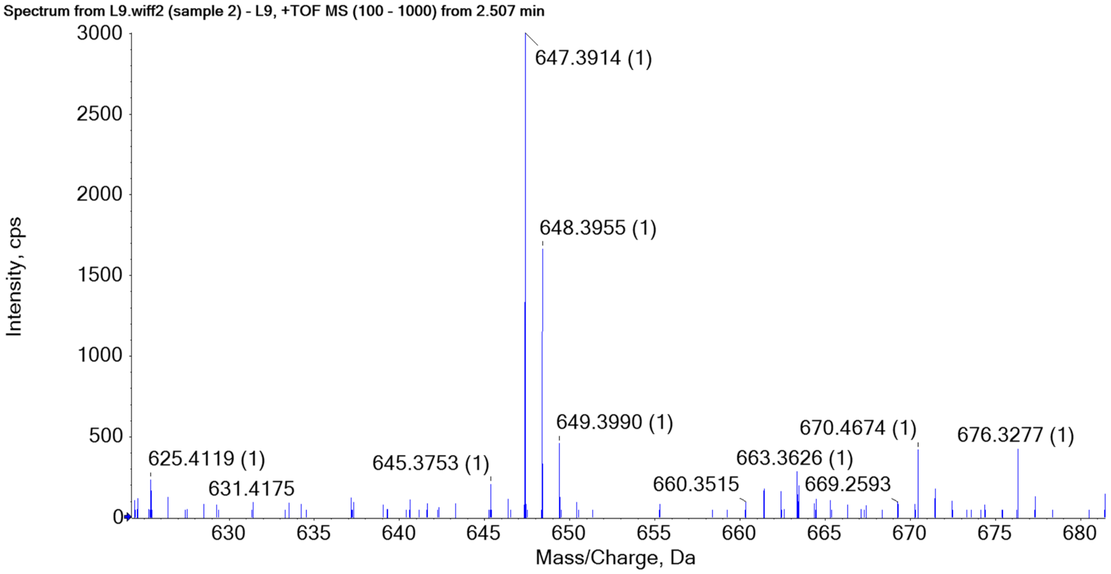

Formula Calculator Results

Measured m/z	Cal m/z	Error(mmu)	Error(ppm)	Ion Formula	Ion
647.3914	647.3918	-0.4	-0.7	C38H56O7Na	[M+Na]+

Figure S6. HR-ESIMS spectrum of 7

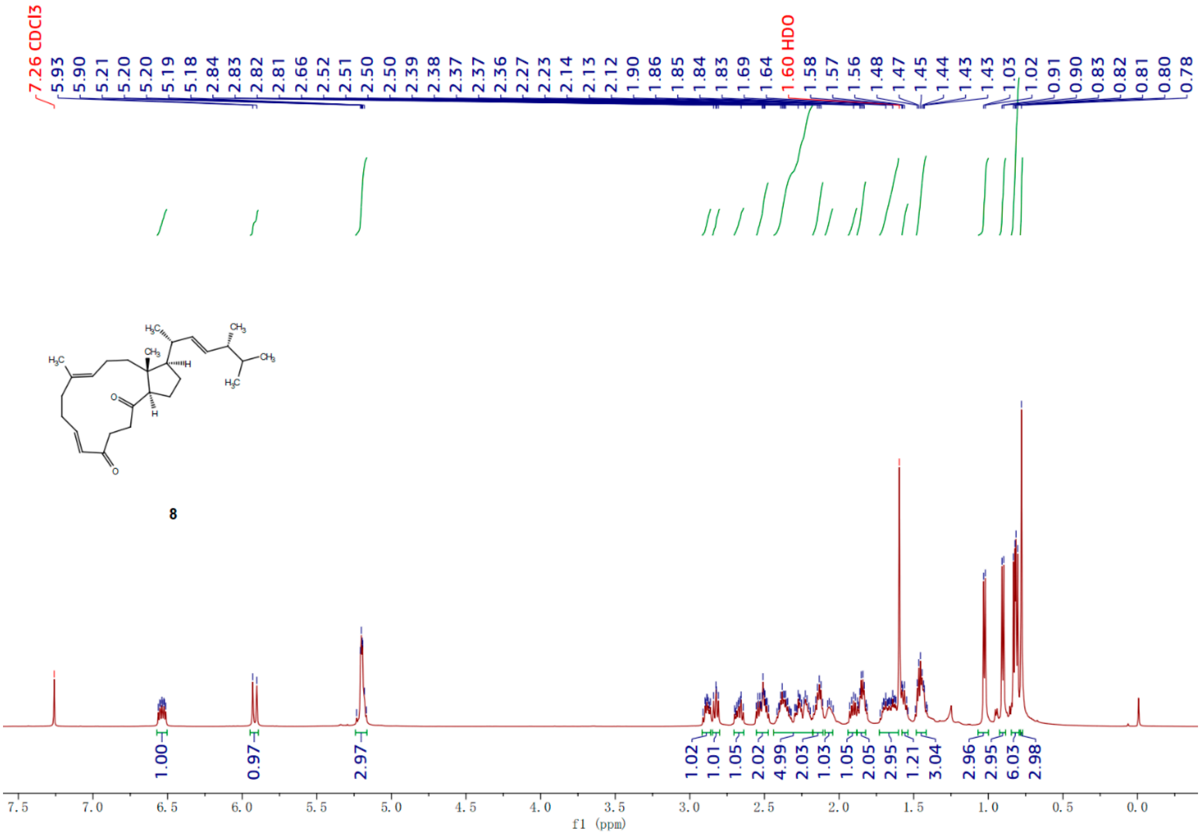

Figure S7. ^{1}H NMR spectrum of 8 (600 MHz, Chloroform-*d*)

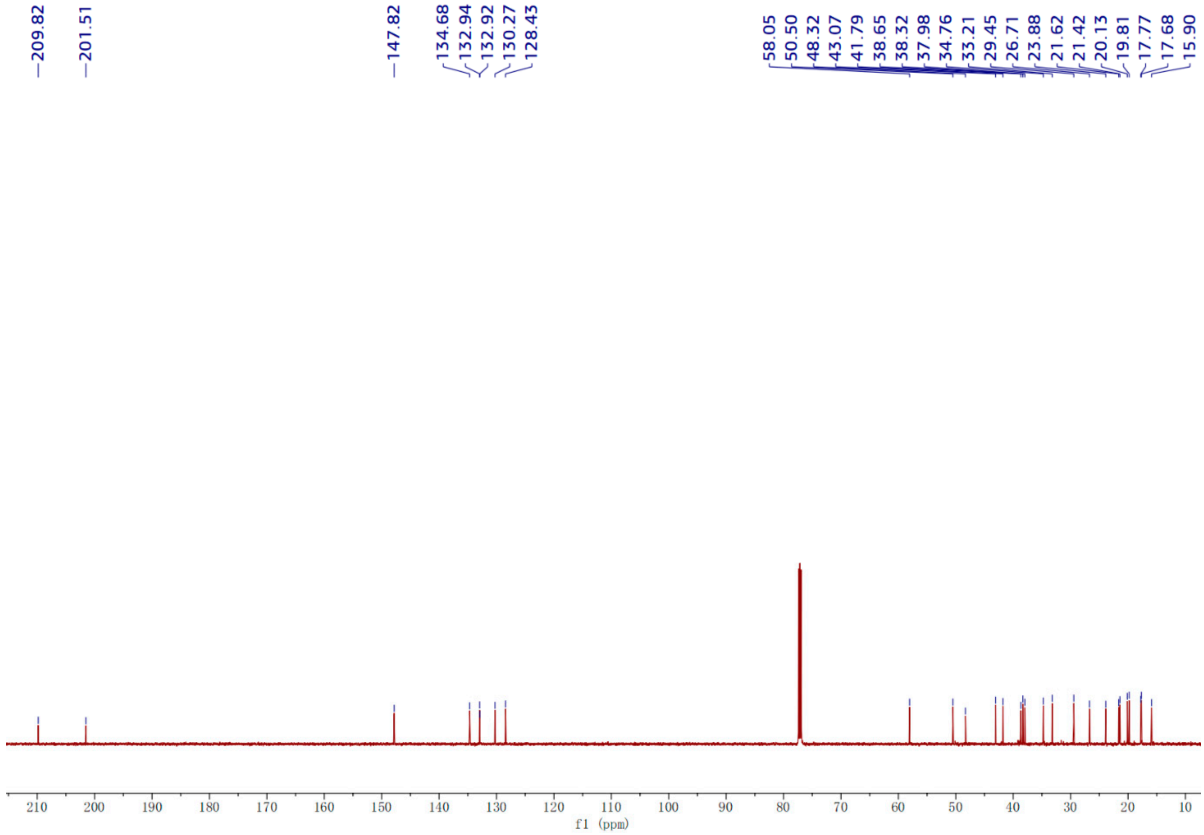

Figure S8. ^{13}C NMR spectrum of 8 (150 MHz, Chloroform-*d*)

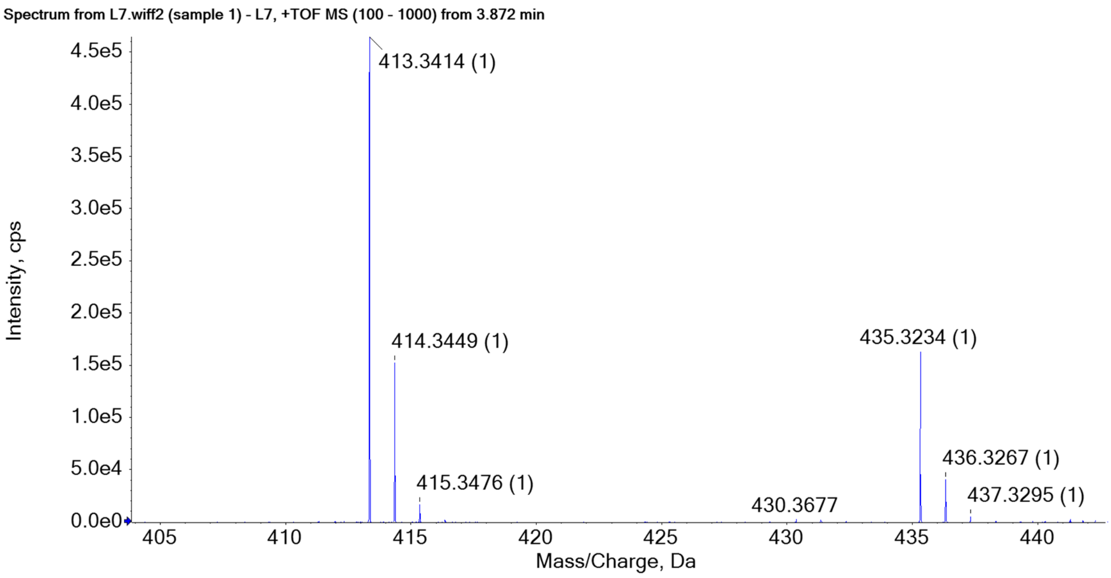

Formula Calculator Results

Measured m/z	Cal m/z	Error(mmu)	Error(ppm)	Ion Formula	Ion
413.3414	413.3414	0	0	C28H45O2	[M+H]+
435.3234	435.3234	0	0	C28H44O2Na	[M+Na]+

Figure S9. HR-ESIMS spectrum of 8

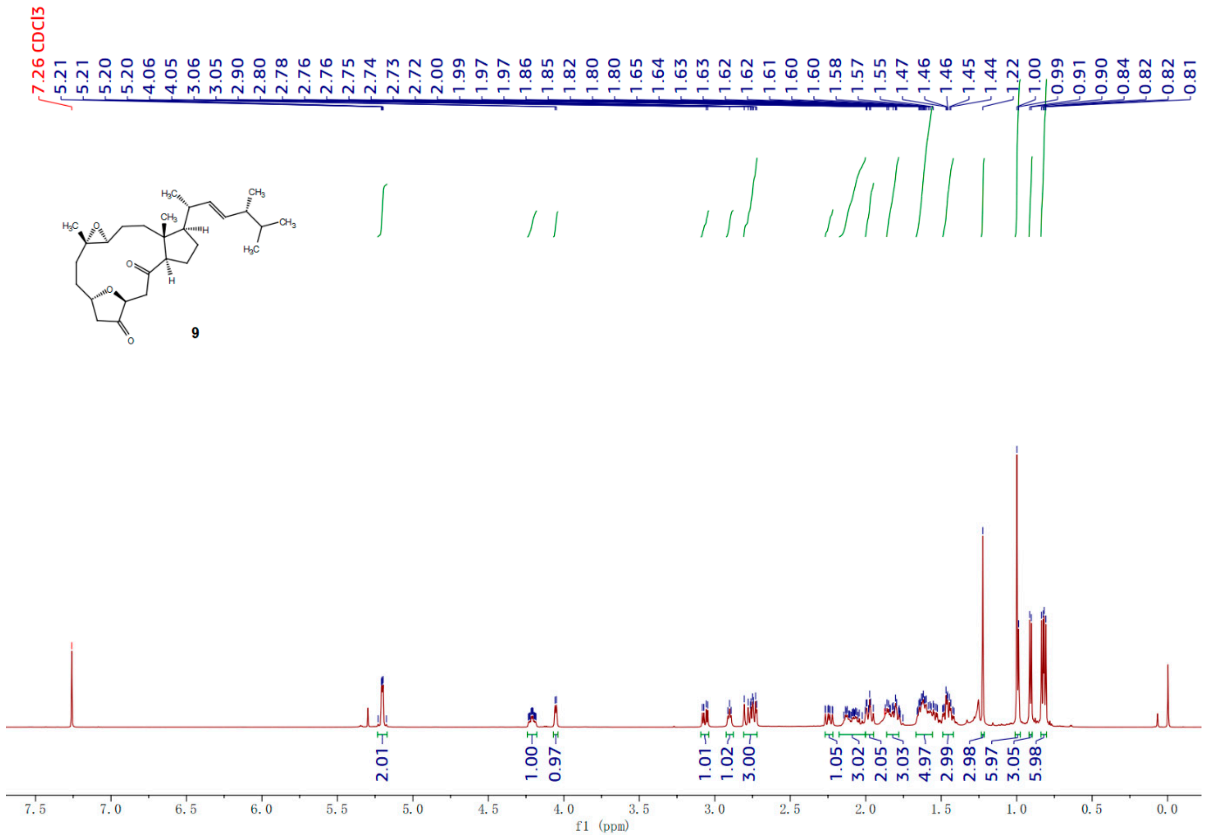

Figure S10. ^{1}H NMR spectrum of 9 (600 MHz, Chloroform-*d*)

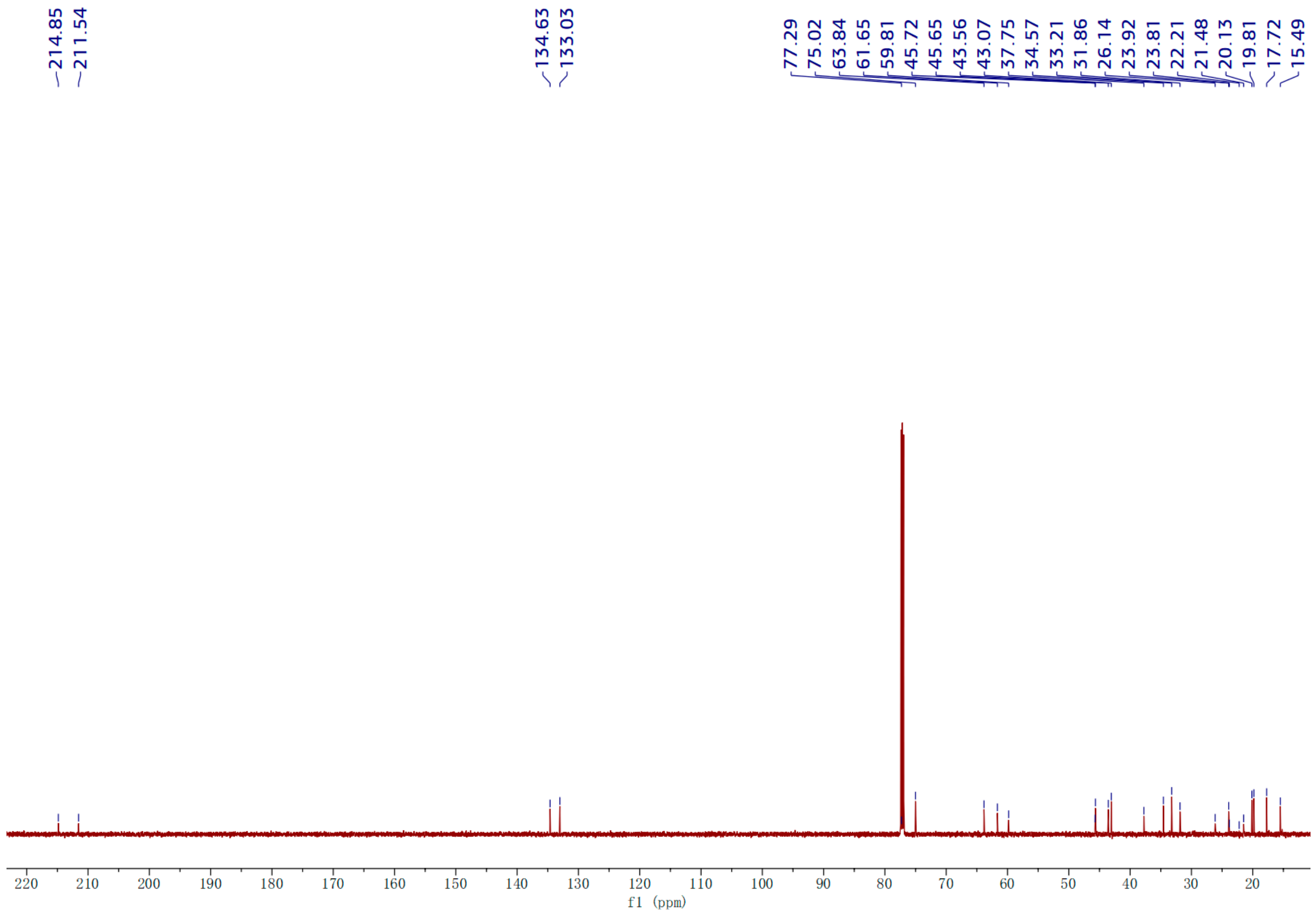

Figure S11. ^{13}C NMR spectrum of 9 (150 MHz, Chloroform-*d*)

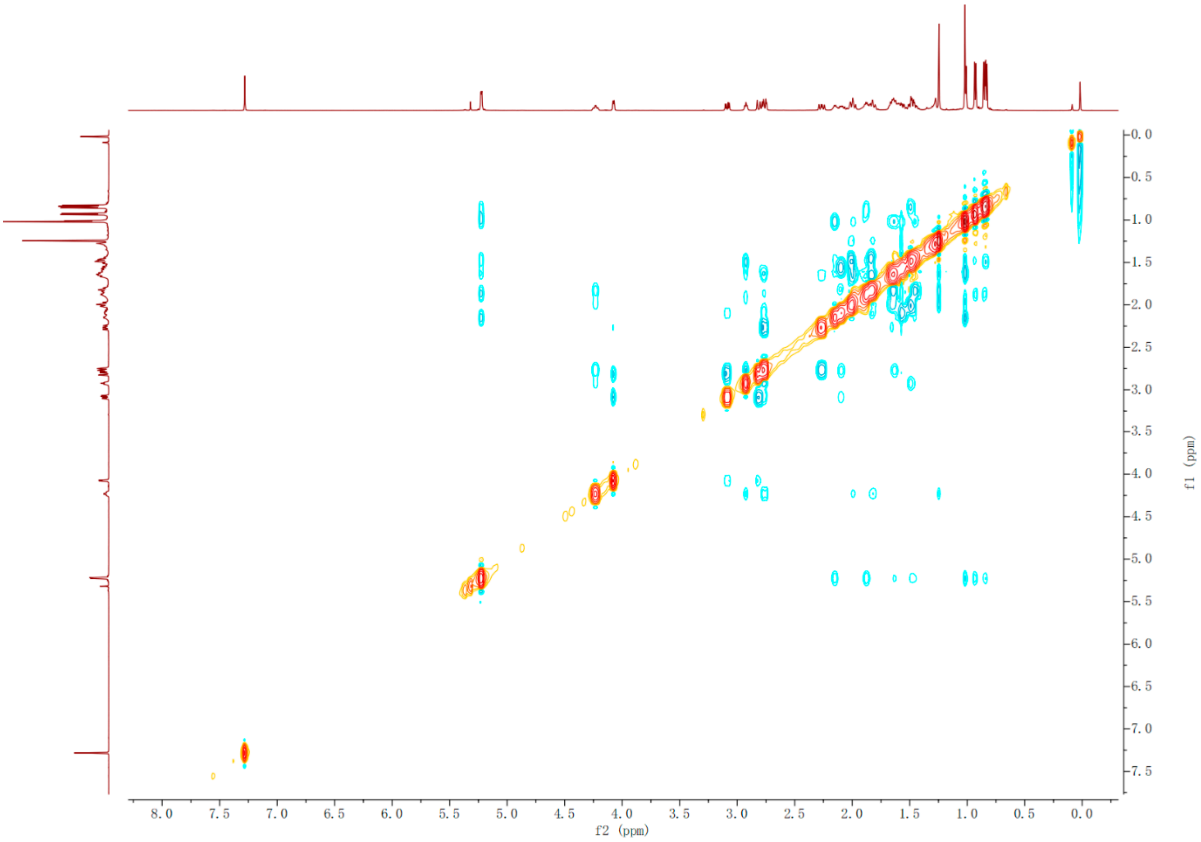

Figure S12. NOESY spectrum of 9 (600 MHz,600 MHz, Chloroform-*d*)

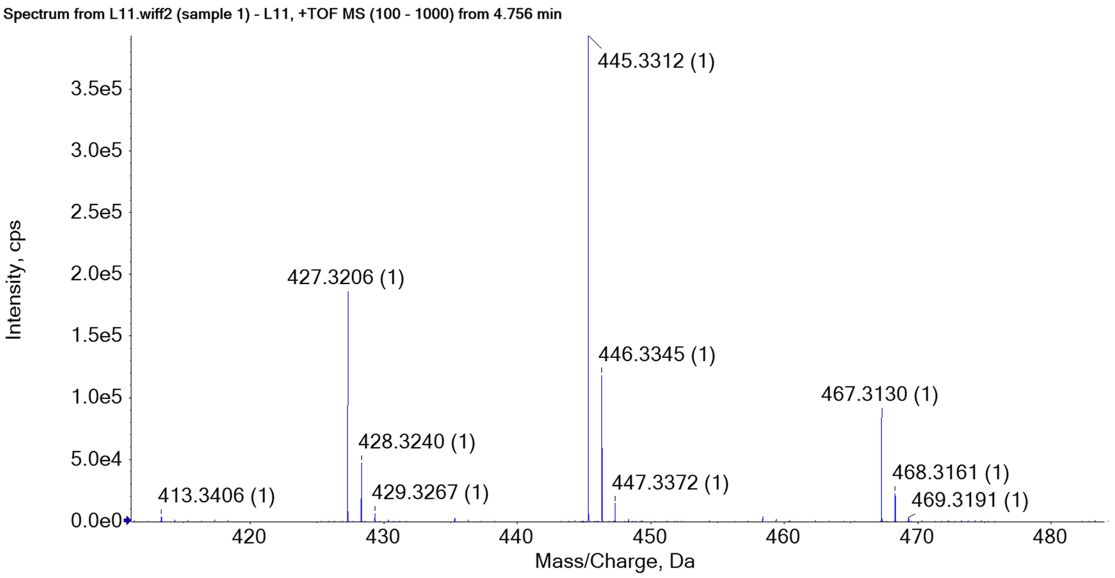

Formula Calculator Results

Measured m/z	Cal m/z	Error(mmu)	Error(ppm)	Ion Formula	Ion
445.3312	445.3312	0	0	C28H45O4	[M+H]+
467.3130	467.3132	-0.2	-0.4	C28H44O4Na	[M+Na]+
427.3206	427.3207	-0.1	-0.3	C28H43O3	[M+H-H2O]+

Figure S13. HR-ESIMS spectrum of 9

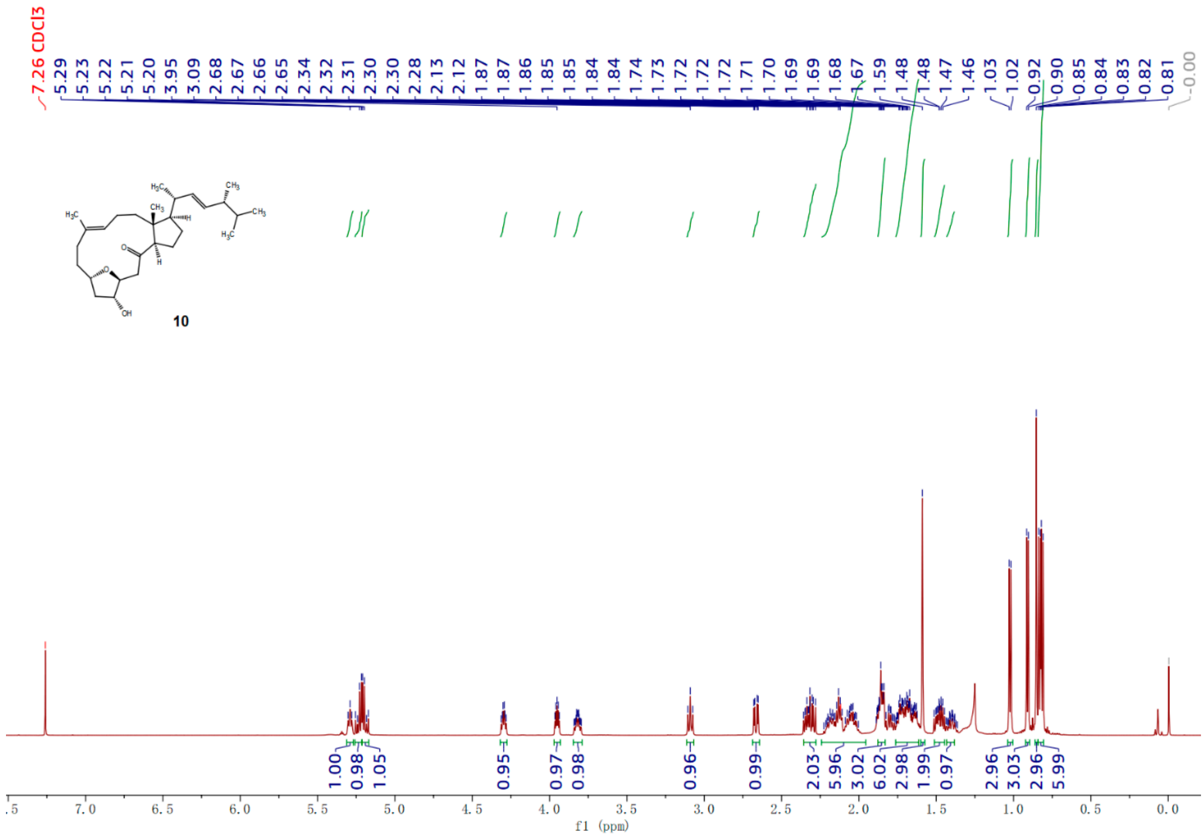

Figure S14. ^{1}H NMR spectrum of 10 (600 MHz, Chloroform-*d*)

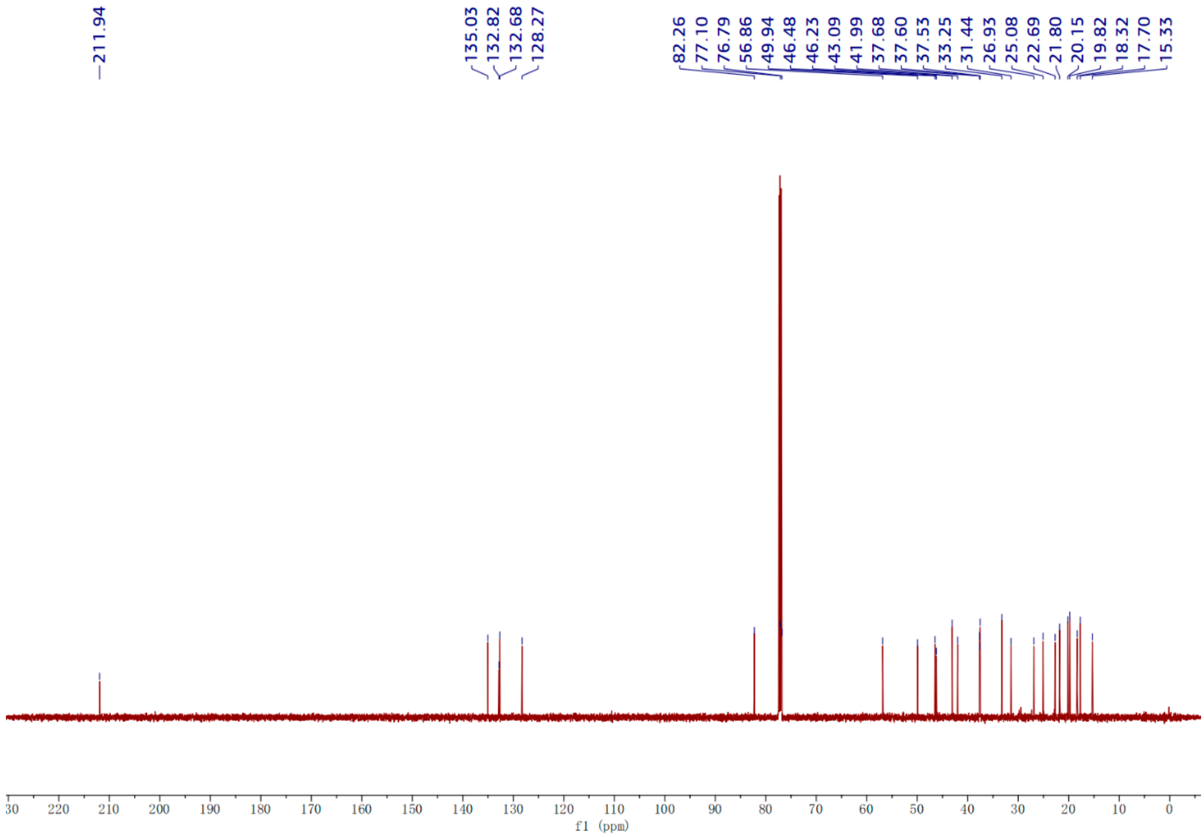

Figure S15. ^{13}C NMR spectrum of 10 (150 MHz, Chloroform-*d*)

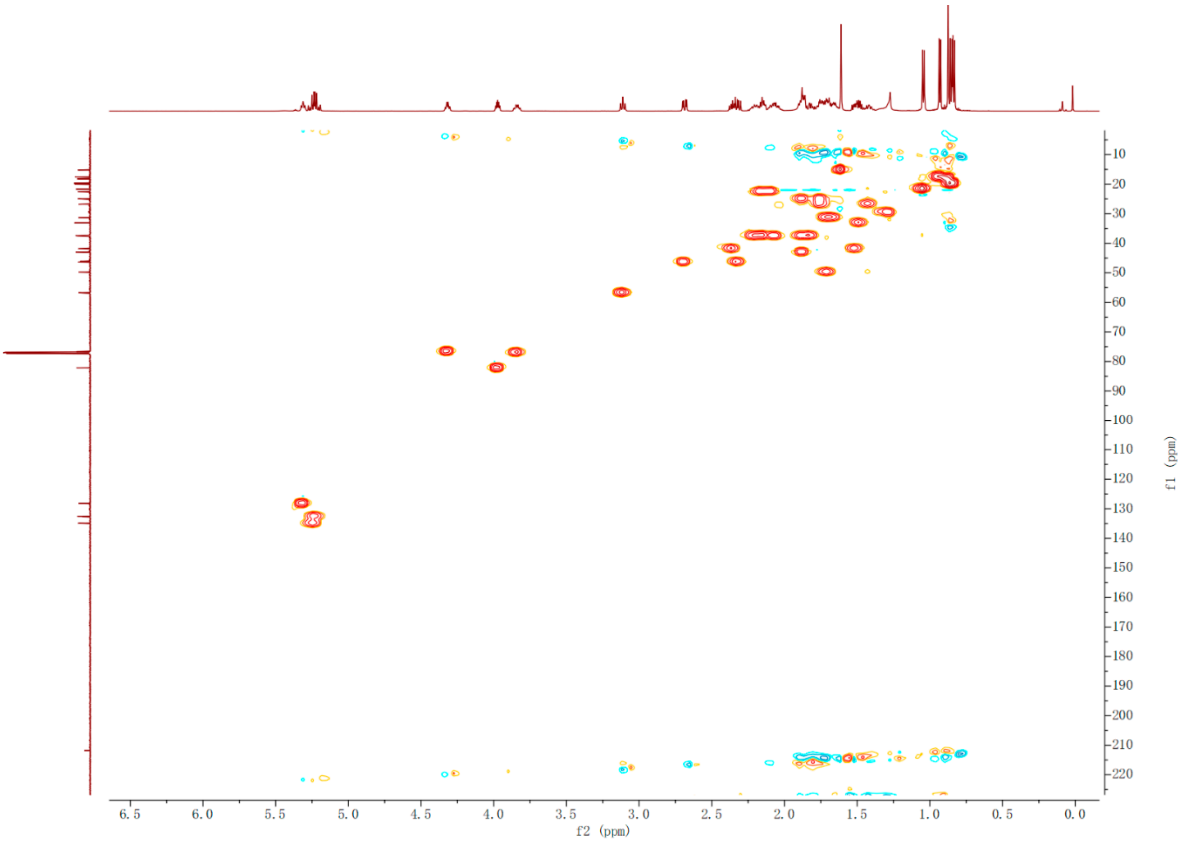

Figure S16. HSQC spectrum of 10 (600 MHz,150 MHz, Chloroform-*d*)

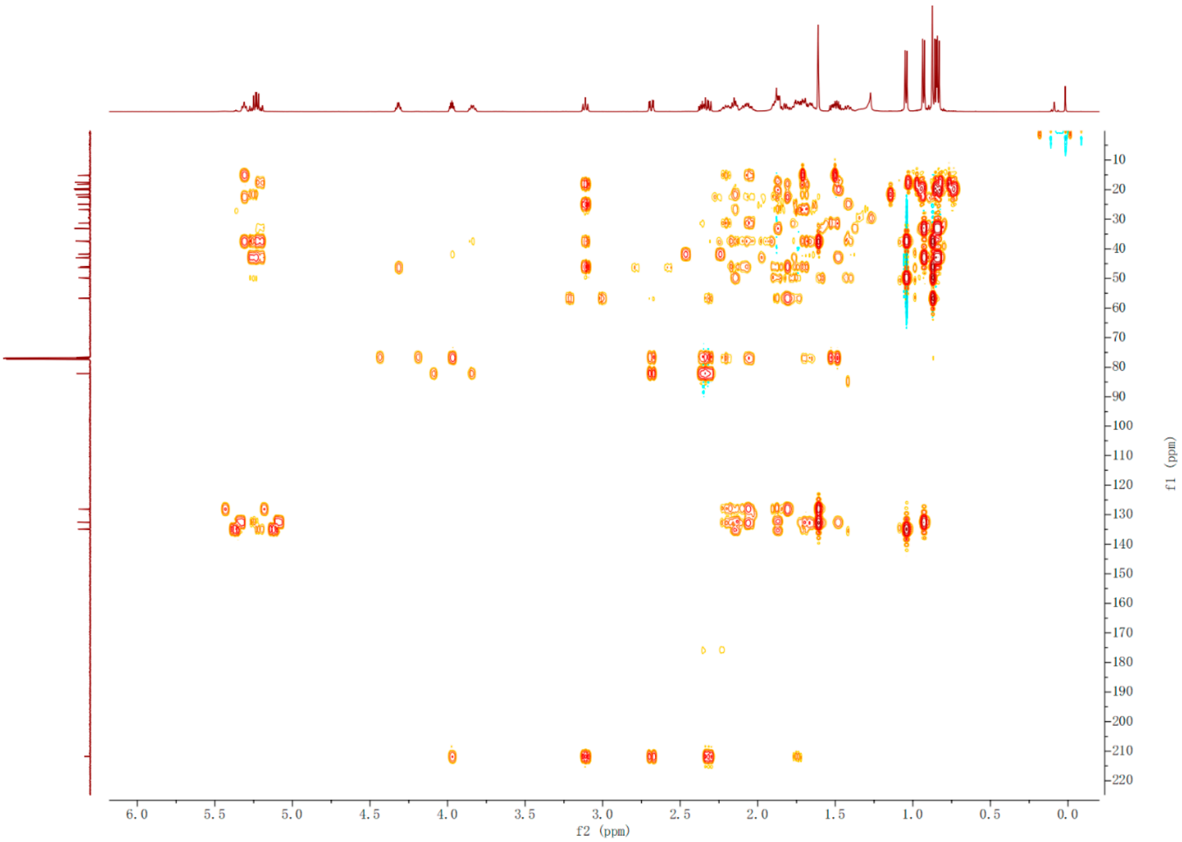

Figure S17. HMBC spectrum of 10 (600 MHz,150 MHz, Chloroform-*d*)

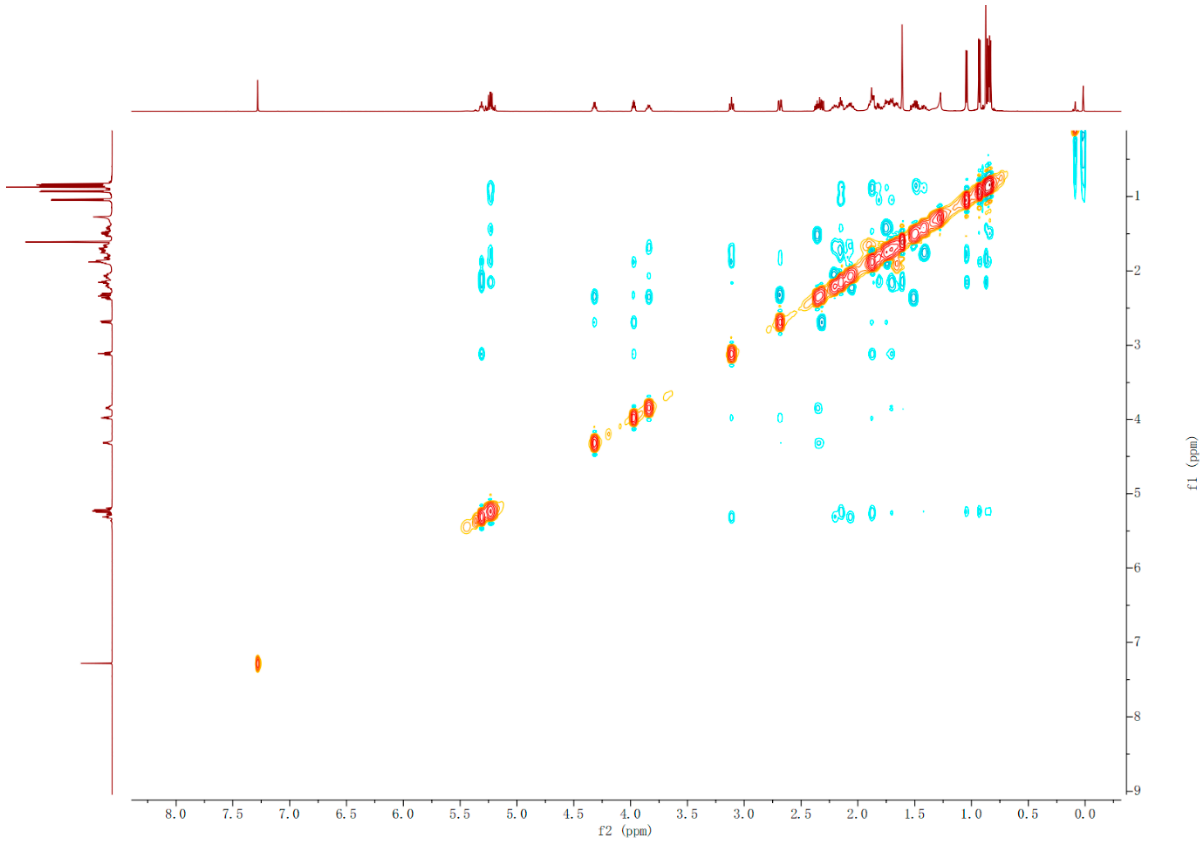

Figure S18. NOESY spectrum of 10 (600 MHz, 600MHz, Chloroform-*d*)

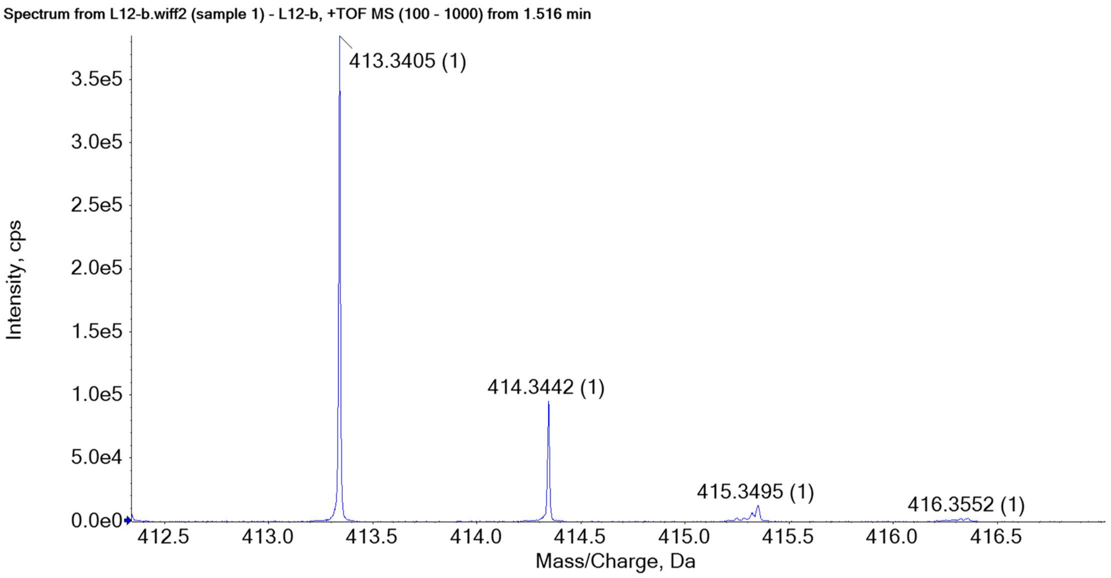

Formula Calculator Results

Measured m/z	Cal m/z	Error(mmu)	Error(ppm)	Ion Formula	Ion
413.3405	413.3415	-1.0	-2.3	C28H45O2	[M+H-H2O]+

Figure S19. HR-ESIMS spectrum of 10

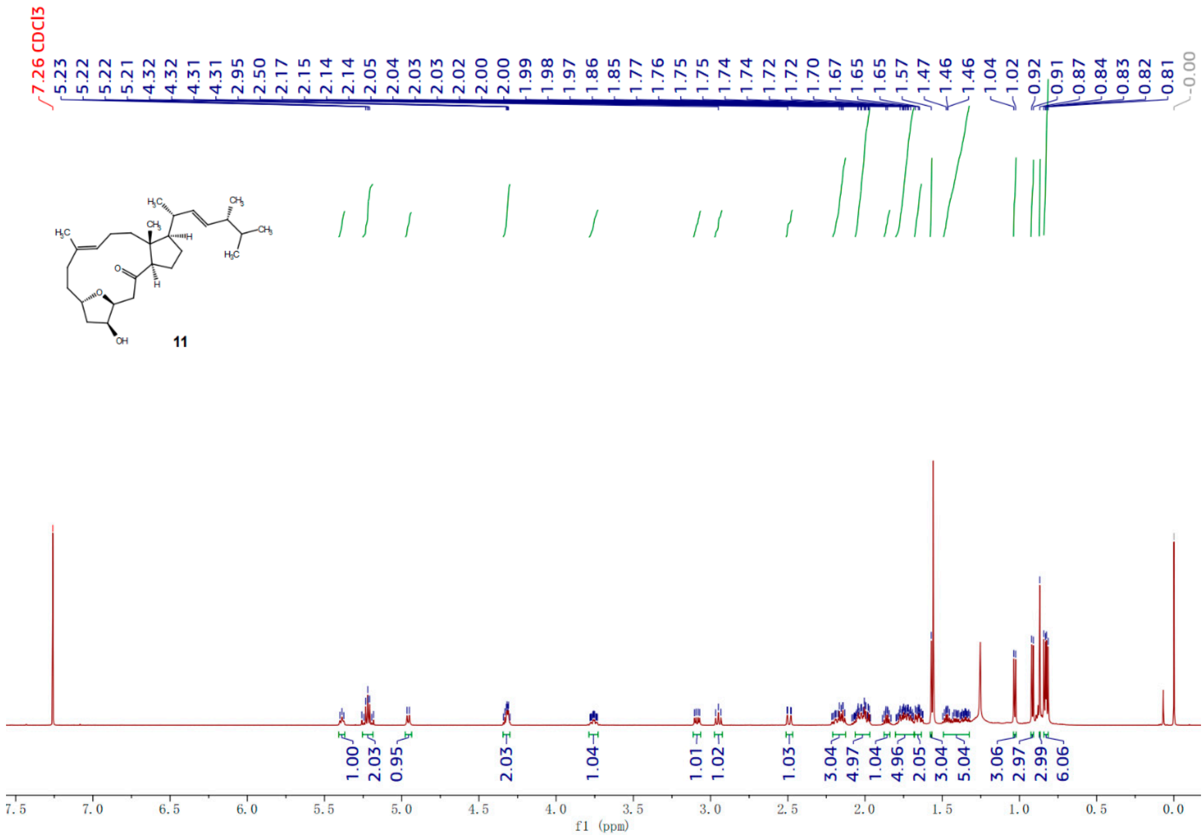

Figure S20. ^{1}H NMR spectrum of 11 (600 MHz, Chloroform-*d*)

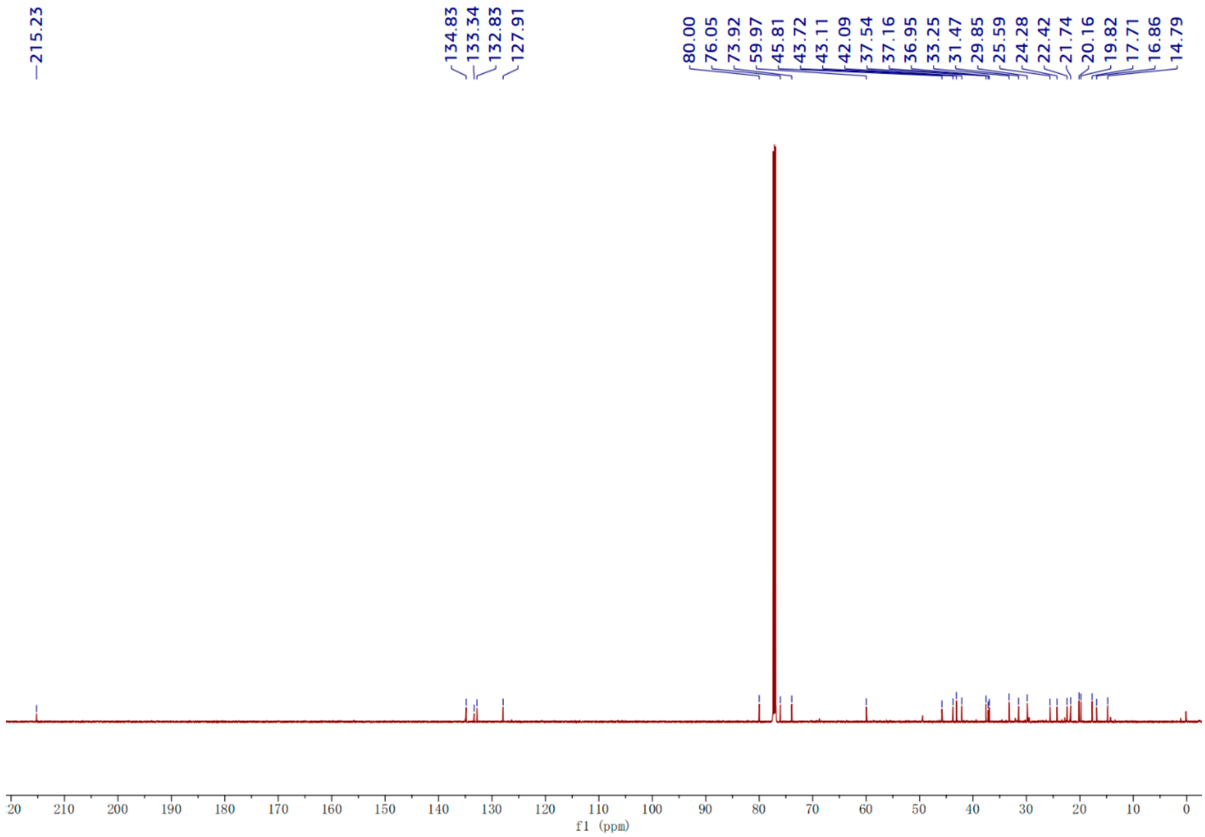

Figure S21. ^{13}C NMR spectrum of 11 (150 MHz, Chloroform-*d*)

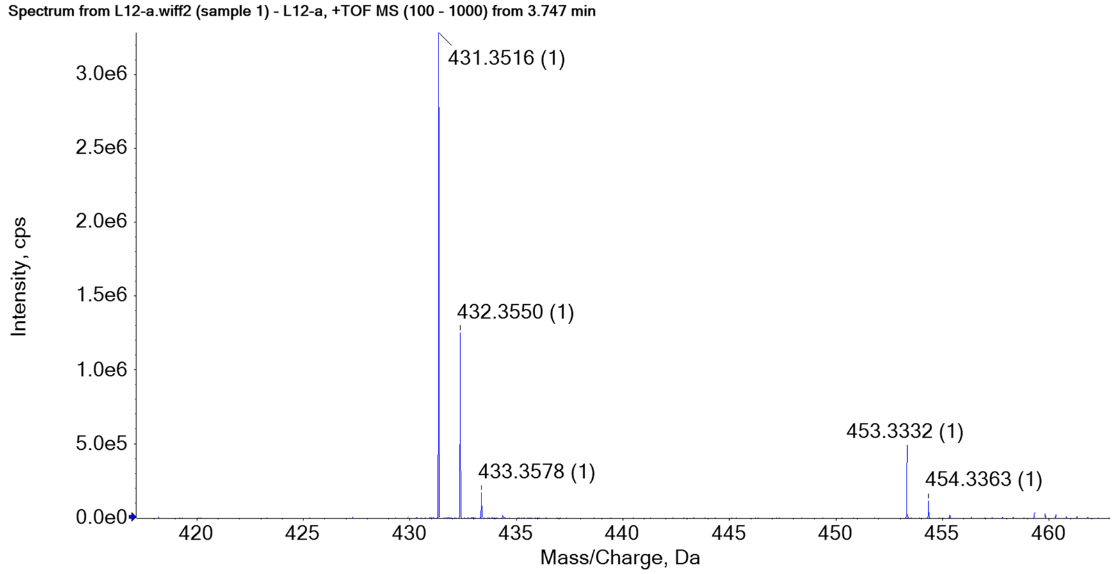

Formula Calculator Results

Measured m/z	Cal m/z	Error(mmu)	Error(ppm)	Ion Formula	Ion
431.3516	431.3520	-0.4	-0.9	C28H47O3	[M+H]+
453.3332	453.3339	-0.7	-1.6	C28H46O3Na	[M+Na]+

Figure S22. HR-ESIMS spectrum of 11

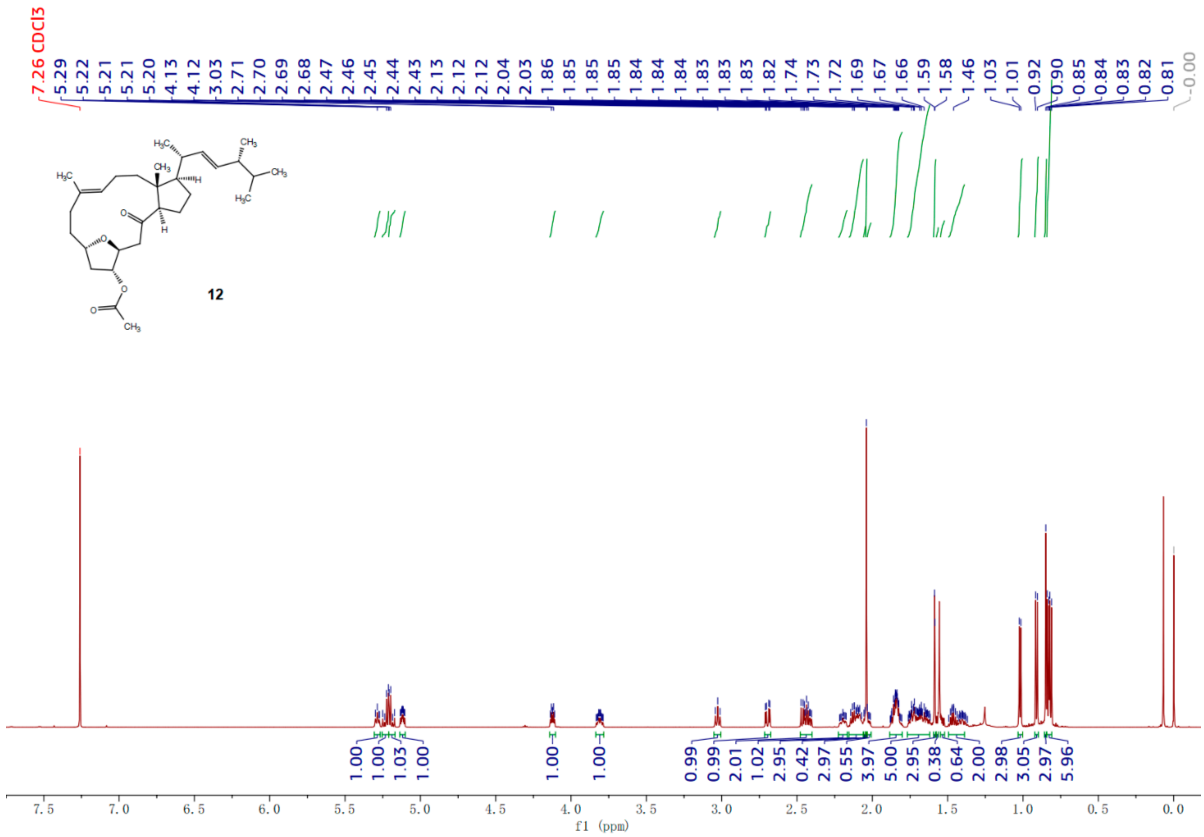

Figure S23. ^{1}H NMR spectrum of 12 (600 MHz, Chloroform-*d*)

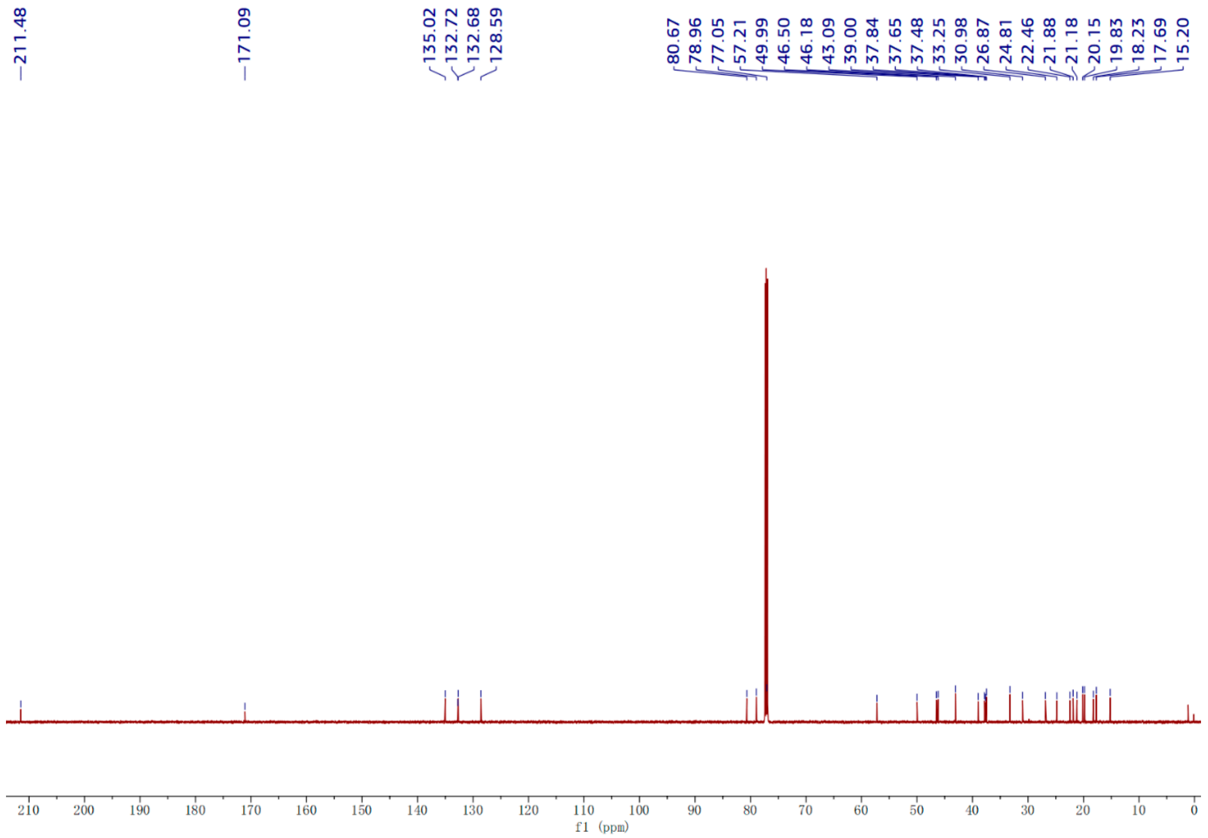

Figure S24. ^{13}C NMR spectrum of 12 (150 MHz, Chloroform-*d*)

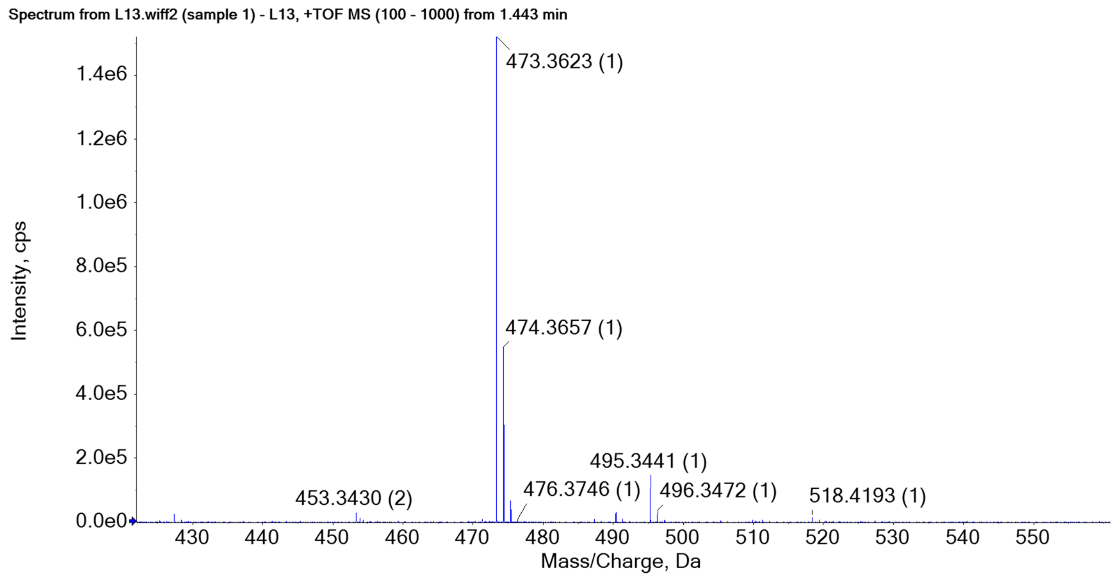

Formula Calculator Results

Measured m/z	Cal m/z	Error(mmu)	Error(ppm)	Ion Formula	Ion
473.3623	473.3626	-0.3	-0.5	C30H49O4	[M+H]+
495.3441	495.3445	-0.4	-0.8	C30H48O4Na	[M+Na]+

Figure S25. HR-ESIMS spectrum of 12

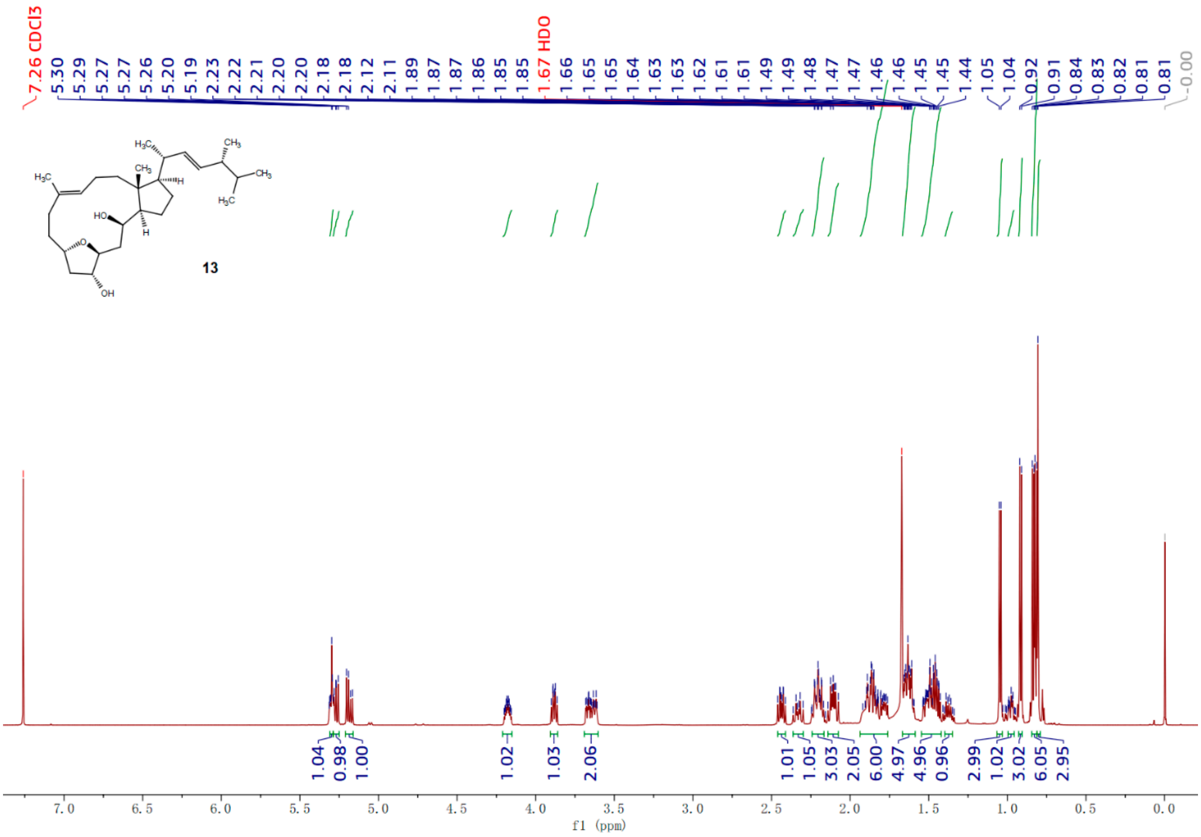

Figure S26. ^{1}H NMR spectrum of 13 (600 MHz, Chloroform-*d*)

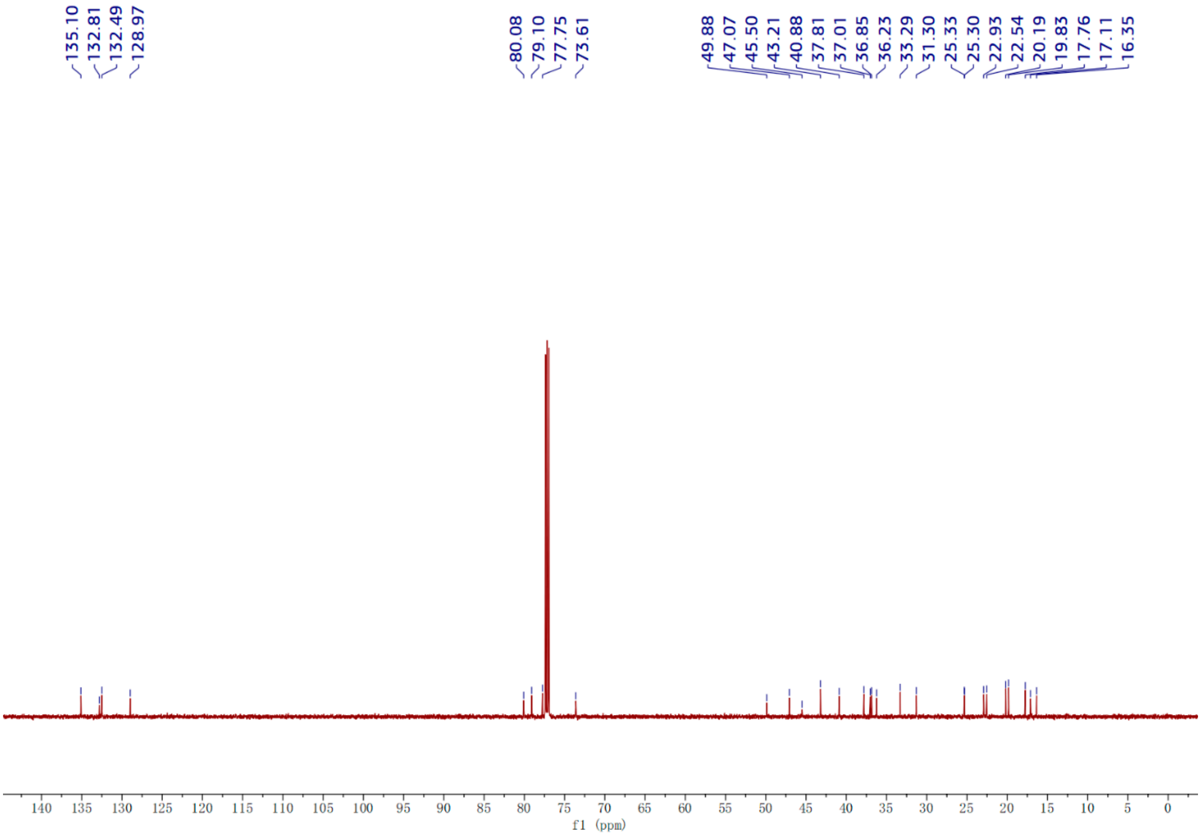

Figure S27. ^{13}C NMR spectrum of 13 (150 MHz, Chloroform-*d*)

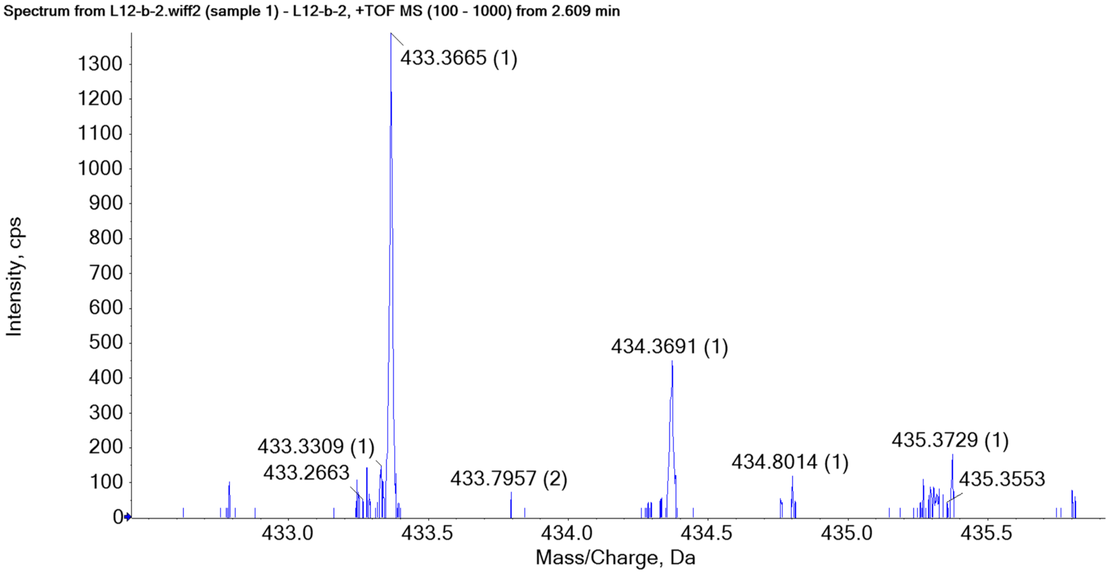

Formula Calculator Results

Measured m/z	Cal m/z	Error(mmu)	Error(ppm)	Ion Formula	Ion
433.3665	433.3676	-1.1	-2.5	C28H49O3	$[M+H]^+$

Figure S28. HR-ESIMS spectrum of 13

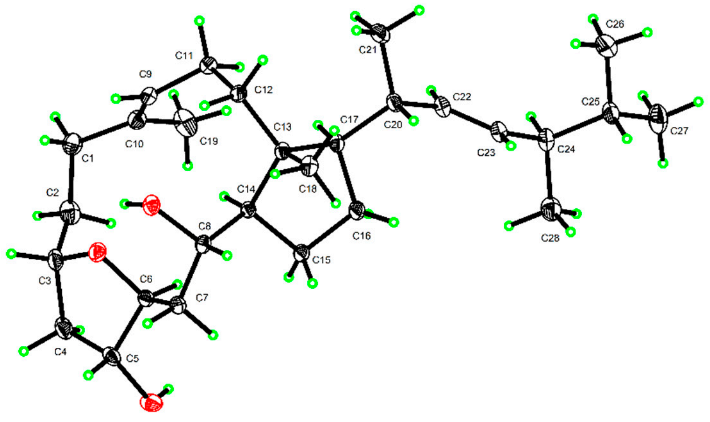

Table 1 Crystal data and structure refinement for L13.

Identification code	L13
Empirical formula	$C_{28}H_{48}O_3$
Formula weight	432.66
Temperature/K	170(2)
Crystal system	monoclinic
Space group	C2
a/Å	32.6594(8)
b/Å	6.6574(2)
c/Å	12.7392(3)
α/°	90
β/°	106.2330(10)
γ/°	90
Volume/Å^3	2659.42(12)
Z	4
ρ_{calc}g/cm^3	1.081
μ/mm^{-1}	0.521
F(000)	960.0
Crystal size/mm^3	0.419 × 0.05 × 0.019
Radiation	CuKα (λ = 1.54178)
2Θ range for data collection/°	5.636 to 150.002
Index ranges	$-40 \le h \le 36$, $-7 \le k \le 8$, $-15 \le l \le 15$
Reflections collected	20529
Independent reflections	5343 [R_{int} = 0.0393, R_{sigma} = 0.0304]
Data/restraints/parameters	5343/1/288
Goodness-of-fit on F^2	1.040
Final R indexes [I>=2σ (I)]	R_1 = 0.0403, wR_2 = 0.1048
Final R indexes [all data]	R_1 = 0.0436, wR_2 = 0.1075
Largest diff. peak/hole / e Å^{-3}	0.23/-0.15
Flack parameter	0.13(8)

Figure S29. Single-crystal X-ray diffraction (SCXRD) data for 13
